# Complete Electroanatomic Imaging of the Diastolic Pathway Is Associated With Improved Freedom From Ventricular Tachycardia Recurrence

**DOI:** 10.1161/CIRCEP.120.008651

**Published:** 2020-07-28

**Authors:** Alexios Hadjis, Antonio Frontera, Luca Rosario Limite, Caterina Bisceglia, Ludovica Bognoni, Luca Foppoli, Felicia Lipartiti, Gabriele Paglino, Andrea Radinovic, Georgio Tsitsinakis, Federico Calore, Paolo Della Bella

**Affiliations:** 1 Arrhythmology Department (A.H., A.F., L.R.L., C.B., L.F., F.L., G.P, A.R., G.T., P.D.B.), IRCCS San Raffaele Hospital, Milan, Italy.; 2 University of Medicine (L.B.), IRCCS San Raffaele Hospital, Milan, Italy.; 3 Division of Cardiology, Hôpital du Sacré-Coeur de Montréal, University of Montreal, Quebec, Canada (A.H.).; 4 Abbott Medical Italy, Sesto San Giovanni, Milan (F.C.).

**Keywords:** ablation, catheter ablation, mapping, recurrence, ventricular tachycardia

## Abstract

The development of multielectrode mapping catheters has expanded the spectrum of mappable ventricular tachycardias (VTs). Full diastolic pathway recording has been associated with a high rate of VT termination during radiofrequency ablation as well as noninducibility at study end. However, the role of diastolic pathway mapping on VT recurrence has yet to be clearly elucidated. We aimed to explore the role of complete diastolic pathway activation mapping on VT recurrence.

**Methods:**

Eighty-five consecutive patients who underwent VT ablation guided by high-density mapping were enrolled. During activation mapping, the presence of electrical activity in all segments of diastole defined the evidence of having had recorded the whole diastolic interval. Patients were categorized as having recorded the full diastolic pathway, partial diastolic pathway, or no diastolic pathway map performed. Recurrences of VT were defined as appropriate implantable cardioverter defibrillator therapies or on the basis of ECG-documented arrhythmia.

**Results:**

Eighty-five patients were included. Complete recording of the diastolic pathway was achieved in 36/85 (42.4%) patients. Partial recording of the diastolic pathway of the clinical VT was achieved in 24/85 (28.2%) patients. No recording of the diastolic pathway of the clinical VT was feasible in 25/85 patients (29.4%). At a mean of 12.8 months, freedom from VT recurrence was 67% in the overall cohort. At a mean of 12.8 months, freedom from VT recurrence was 88%, 50%, and 55% in patients who had full diastolic activity recorded, partial diastolic activity recorded, or underwent substrate modification, respectively; the observed differences were statistically significant (*P*=0.02).

**Conclusions:**

Mapping of the entire diastolic pathway was associated with a higher freedom from VT recurrence as compared with partial diastolic pathway recording and substrate modification. The use of multielectrode mapping catheters in recording diastolic activity may help predict those VTs employing intramural circuits and further optimize ablation strategies.

## Abstract 2

Supplemental Digital Content is available in the text.

**What Is Known?**

- Despite advancements in mapping catheters, as well as electroanatomic mapping systems, success rates of ventricular tachycardia (VT) ablation have plateaued.
- Full diastolic pathway mapping of VT can be performed in a short amount of time and has been associated with a high rate of VT termination with radiofrequency in addition to noninducibility at study end.

**What the Study Adds?**

- Mapping of the full diastolic pathway is associated with a higher freedom from VT recurrence as compared with partial mapping or substrate modification.
- Full diastolic pathway recording of VT can be achieved in a large proportion of patients.
- Patients in whom incomplete diastolic activity is tracked on the endocardial surface, epicardial mapping should be considered as a first line indication to identify the missing link to record the entire diastolic pathway.

Catheter ablation of ventricular tachycardia (VT) is increasingly performed worldwide,^1,2^ however, success rates of ablation have plateaued.^3^ While numerous approaches to ablation have been developed, particularly in the domain of substrate modification,^4^ the advancements in multielectrode mapping catheters have expanded the spectrum of mappable VTs. The increased number of electrodes, in addition to extent of the recording area and electrode orientation, have proven useful in creating fast and reliable activation maps during VT. Most importantly, in cases of hemodynamically unstable VT, these maps can be produced in a reasonable time frame.^5^ Full diastolic pathway recording has been associated with a high rate of VT termination during radiofrequency ablation as well as noninducibility at study end.^5^ However, the role of diastolic pathway mapping on VT recurrence has yet to be clearly elucidated. We sought to explore how frequently full electroanatomic imaging of the diastolic pathway mapping can be achieved and how this translates to clinical outcome.

## Methods

The data that support the findings of this study are available from the corresponding author upon reasonable request.

### Study Population

A retrospective analysis was performed in patients referred to our institution for VT ablation from December 2017 to June 2019 whom underwent ablation with the EnSite Precision Cardiac Mapping System (Abbott, MN), guided by high-density mapping with the Grid mapping catheter (GMC; Advisor HD Grid Mapping Catheter Sensor Enabled, Abbott, MN). Patients with both ischemic cardiomyopathy (ICM) and nonischemic cardiomyopathy (NICM) were included in the study. Only patients with at least 30 seconds of mappable VT were included. Patients with a recent acute coronary syndrome, recent coronary artery bypass grafting, or any history of left ventricular assist device implant were excluded. The study was approved by the institutional review committee, and all patients gave their written informed consent.

### Definitions

VTs induced at the time of ablation were defined as clinical if they matched the arrhythmia captured clinically on 12 lead ECG or, in cases where no ECG was available, they matched the tachycardia cycle length of the implantable cardioverter defibrillator log.

Arrhythmia recurrence was defined as any arrhythmia receiving device-based treatments ATP or shock or any VT episodes assessed at clinical evaluation.

### Workflow

The procedures were performed under general anesthesia, with continuous invasive monitoring of the arterial pressure, arterial oxygen saturation, and acid/base balance, according to our standard protocol as previously described.^6^ In cases where no 12 lead ECG of the clinical VT was available, programmed ventricular stimulation (PVS) with a standard fixed curve quadripolar catheter at the right ventricular apex was performed at study onset under conscious sedation before induction of general anesthesia.

Epicardial mapping and ablation was favoured in patients with a history of previously failed endocardial ablation, myocarditis, or arrhythmogenic right ventricular dysplasia. Epicardial access, in cases where a detailed endocardial mapping during VT failed to reveal the complete diastolic interval, was attempted within the same procedure unless considered inadvisable due to hemodynamic instability or overall unfavourable patient conditions. Epicardial access was not attempted in patients with a previous history of cardiac surgery.

At first, a geometry of the chamber of interest was created with a Flexability Ablation Catheter Sensor Enabled (Abbott, MN); then, a GMC was used to generate the substrate map either during sinus rhythm (SR) or right ventricular pacing in pacing dependent patients. In those patients with cardiac resynchronization therapy devices, left ventricular pacing was turned off. Voltage map data were achieved using the GMC in the HD Wave bipolar configuration and the Best Duplicate Algorithm as previously described.^5^ Isochronal late activation mapping, where SR propagation was displayed with 8 equally distributed isochrones of activation, was routinely performed to investigate the presence isochronal crowding (>2 isochrones within a 1 cm radius) and conduction delay of propagation. SR activation map timing was set at the termination of the latest electrogram deflection at any given point. Subsequently, PVS (up to 4 extrastimuli delivered at the right ventricular apex and multiple left ventricular sites) was performed to induce VT.

### Diastolic Pathway Mapping

Activation mapping was attempted on all induced arrhythmias by sequentially positioning the GMC, starting at sites showing the maximum conduction delay at baseline SR mapping. This was guided by visual identification of conduction slowing, based on isochronal late activation mapping, as previously described,^7^ performed during SR. The window of interest was then opened from the termination of the first QRS to the onset of the second QRS of the VT cycle, to define the diastolic interval. The diastolic interval was then divided into 3 main segments, as defined by the timing of the first deflection of the electrogram recorded at any bipole during the VT diastolic interval:

1. 0% to 35% of the diastolic interval defined entrance
2. 36% to 65% of the diastolic interval defined isthmus
3. 66% to 100% of the diastolic interval defined exit

According to the extent of the electrogram recording during mapping, patients were categorized as either having recorded the full diastolic pathway, partial diastolic pathway, or no diastolic pathway of the clinical VT.

**Figure 1.**
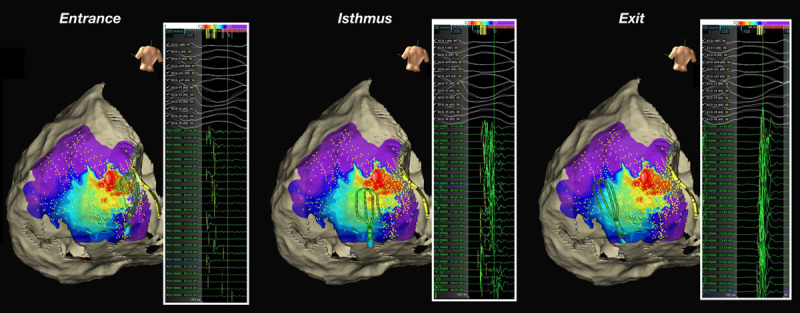
**Ventricular tachycardia (VT) with the full diastolic pathway identified on epicardium with respective electrograms.** As the window of interest is set at the diastolic phase, the diastolic pathway is demonstrated with sequential mappings from entry, through the isthmus, and to exit. First deflection annotation timing is marked by yellow bars for the respective electrograms.

Full diastolic pathway mapping was defined as having recorded the presence of electrical activity in all segments, bridging each QRS to the next one (Figure 1).

Partial diastolic pathway mapping was defined as having recorded electrical activity in at least 1 of the 3 diastolic segments.

No diastolic pathway mapping indicated that no electrograms were seen in the diastolic window despite detailed mapping of the VT and were defined as the SR substrate modification group.

### Ablation

In cases where electrical activity was present throughout the entire diastolic interval, a fully functional imaging of the diastolic pathway was displayed. Using this functional electrical imaging of the reentry, demonstrating both extent and width of the channel, in addition to funnels at the entry and exit, the ablation strategy targeted interruption of the reentrant activation at the narrowest point between the boundaries of the VT channel (Movie in the Data Supplement). A line of ablation was performed at the narrowest site, usually between entrance and isthmus sites.

**Figure 2.**
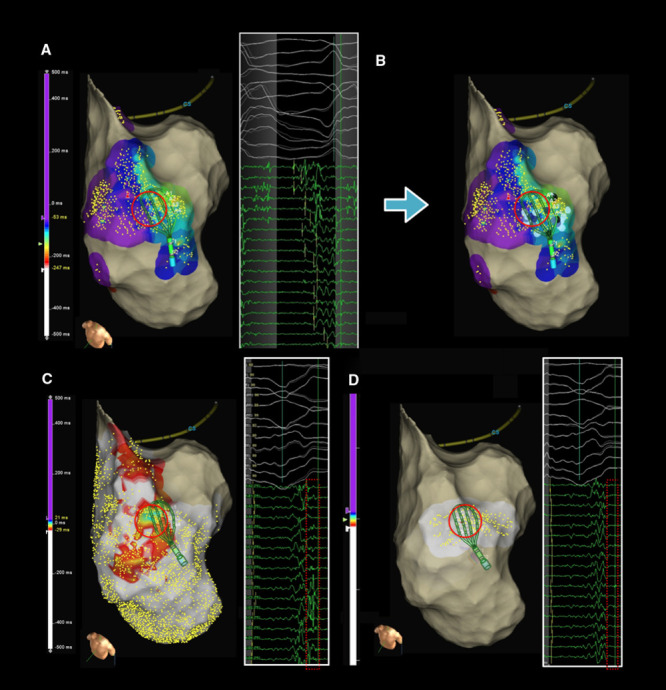
**Ablation strategy.** **A**, Ventricular tachycardia (VT) activation map displaying mid-isthmus to exit activity, encircled in red. **B**, Ablation lesion set targeting diastolic activity. **C**, Near field electrograms during sinus rhythm (SR) mapping at same site of VT reentry before ablation. **D**, Remap illustrates the end point of near field activity abolition following ablation at the site of diastolic activity.

This was performed during VT, or in cases of hemodynamically non tolerated VT, following arrhythmia termination during sinus or paced rhythm by delivering contiguous lesions to organize a transecting line at the described level using the VT activation map as reference. Complete elimination of near field activity at sites involving the VT reentry was confirmed with subsequent remap confirming elimination of near field activity during SR (Figure 2).

**Figure 3.**
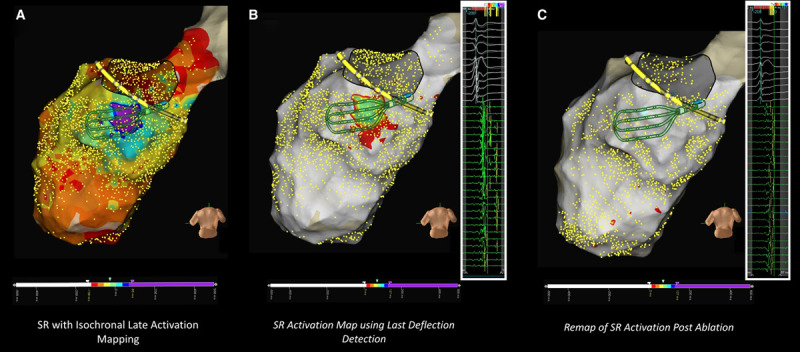
**Substrate modification ablation strategy.** **A**, Isochronal late activation mapping reconstruction of sinus rhythm (SR) activation demonstrating isochronal crowding along the inferobasal left ventricle. **B**, SR activation map demonstrating late potentials at the same site using last deflection detection timing. **C**, Following catheter ablation at site of conduction slowing, remap shows abolition of late potentials.

In cases where the diastolic pathway map was not completed, zones of isochronal crowding, in addition to zones of conduction slowing, visually confirmed with SR local activation time propagation maps, were targeted for ablation until late potential (LPs) were modified or eliminated. LP abolition was routinely confirmed with remapping post ablation (Figure 3).

The power was set to 50 W, the tip temperature read <45°C, the irrigation rate was 17 mL/min, the max impedance drop was 20 Ohms. Ablation lesions were performed for a duration of 60 to 120 seconds with catheter maneuvers performed to maintain catheter stability as guided by dedicated software tools (Automark) of the Ensite Precision Cardiac Mapping System.

**Figure 4.**
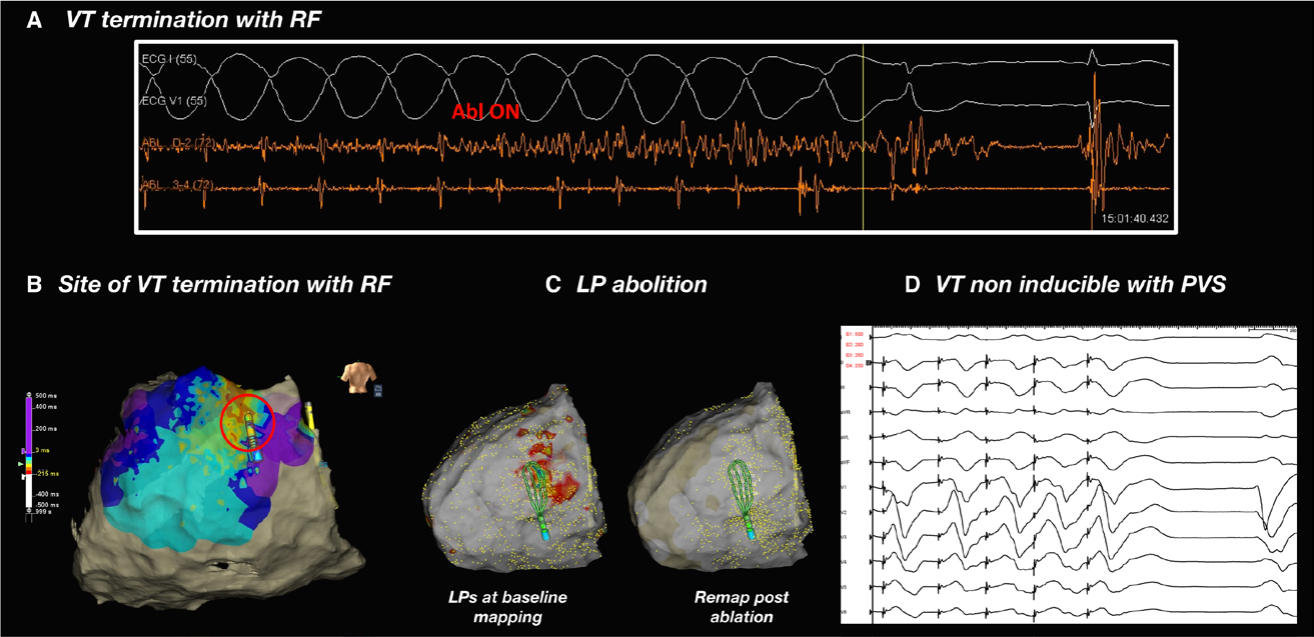
**Ablation end points.** **A**, Termination of ventricular tachycardia (VT) within 3.1 seconds of radiofrequency (RF) and associated catheter position (**B**) at mid isthmus. **C**, Pre and post ablation sinus rhythm (SR) activation maps demonstrating abolition of late potentials (LPs) at site of SR conduction slowing. **D**, Post ablation, full Programmed Ventricular Stimulation (PVS) protocol is performed with VT noninducibility demonstrated.

End points included all of the following: (1) VT termination; (2) elimination of near field activity (ablation continued until absence of near field activity was confirmed at the sites where diastolic activity had been recorded from subsequent remaps performed with the GMC); (3) elimination of LPs at sites of conduction slowing during SR; and (4) VT noninducibility using the full induction protocol used at baseline (Figure 4).

### Follow-Up

All patients were followed at 3-month intervals with remote monitoring or office visits where implantable devices were interrogated and during any symptomatic event. Two experienced electrophysiologists (Drs Frontera and Hadjis) reviewed the stored implantable cardioverter defibrillator electrograms and adjudicated the arrhythmic events. Antiarrhythmic medications were discontinued upon hospital discharge if complete noninducibility was achieved. Implantable cardioverter defibrillator programming, using high rate implantable cardioverter defibrillator therapy cutoff criteria and delayed arrhythmia detection, was performed according to standard protocol.^8^

### Statistical Analysis

The continuous variables are presented as mean±SD (if normally distributed) and median (interquartile range) otherwise; the categorical variables are reported as count (percentage).

To account for the effect of competing risks (ie, death from all causes) on the estimates on the incidences relative to the event of interest (VT recurrences), cumulative incidence functions were computed; Gray test was used for group comparison. A multivariable Fine and Gray proportional subdistribution hazards regression model was built to assess the relationship between the treatment (diastolic mapping—substrate mapping was used as reference category), the potential confounders (gender, age, ejection fraction, ischemic yes/no, amiodarone at discharge, number of VTs induced during the procedure—selected via backward selection) and the event of interest (VT recurrence) over time. All tests were 2-sided, and *P* values below 0.05 were considered statistically significant. R, version 3.6.2, was used for the analyses (packages: tidyverse, survival, survminer, pec, cmprsk, crrstep).

## Results

### Study Population

A total of 85 consecutive patients were enrolled in this study (mean age of 62.0±12.4 years, mean left ventricular ejection fraction of 38.8±12.2%, mean left ventricular ejection fraction excluding arrhythmogenic right ventricular dysplasia, and myocarditis patients of 35.1±10.2) with a total of 147 VTs induced (mean VT CL 373±94 ms). The clinical characteristics of the study population are reported in Table 1. Epicardial access was obtained in 23 (27%) patients. Periprocedural complications included 3 cases of pericarditis, 2 femoral pseudoaneurysms, and 2 cases of cardiac tamponade treated successfully with pericardial drain. In the overall cohort, 18 (21%) of patients were discharged from hospital on amiodarone. Overall mortality was 7% at 18 months.

**Table 1.**
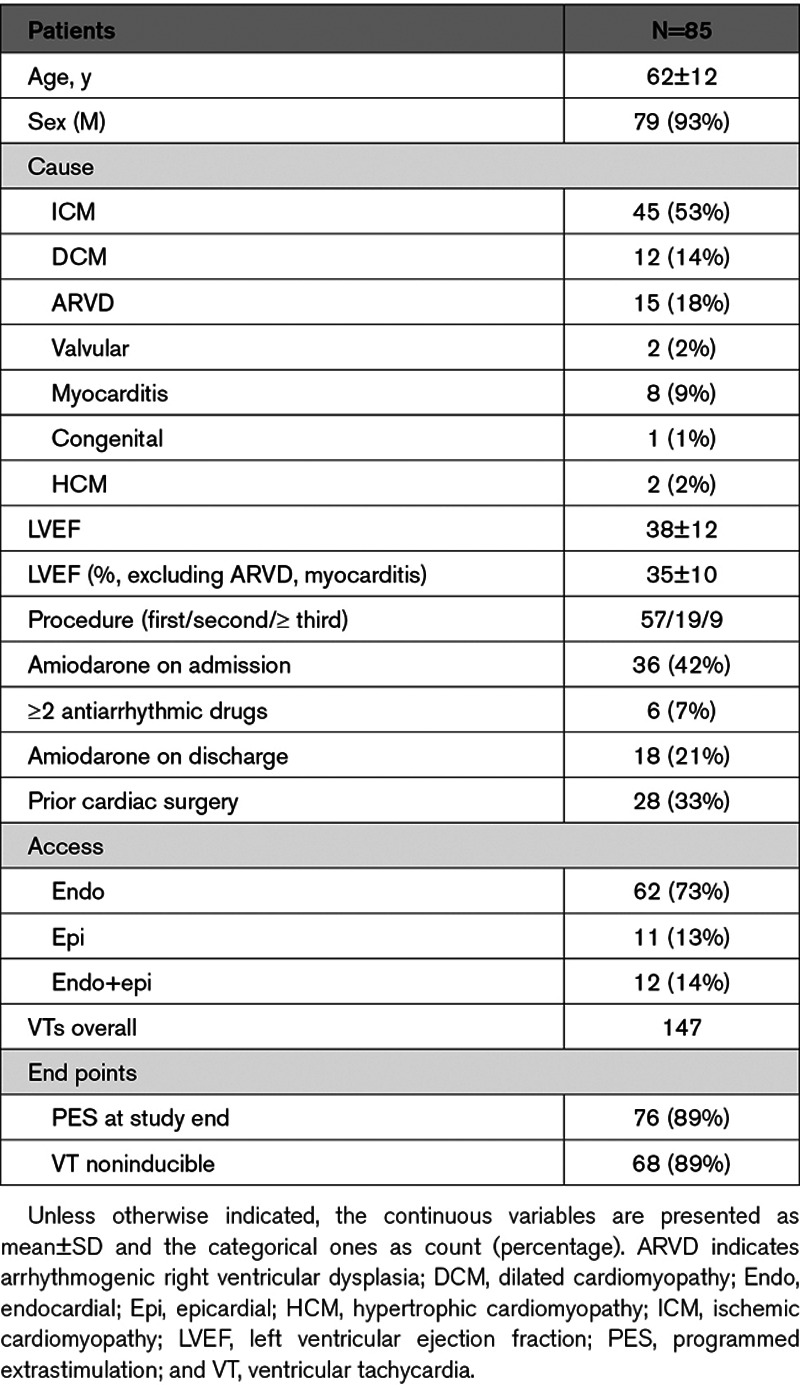
Patient Characteristics and Procedure Details

### Recording of the Diastolic Pathway

Complete recording of the diastolic pathway of the clinical VT was achieved in 36/85 (42%) patients. The full diastolic pathway of the clinical VT was recorded endocardially only in 28/36 patients (78%), epicardially only in 5/36 (14%) patients, and endoepicardially combined access in 3/36 (8%).

Partial recording of the diastolic pathway of the clinical VT was achieved in 24/85 (28.2%) patients. The missing aspect of the diastolic pathway of the clinical VT occurred at the entry site in 18 patients, at the isthmus in 14 patients, and at the exit area in 7 patients. Overall, 6/24 (25%) patients had 2 of 3 segments mapped and 18/24 (75%) had 1 of 3 segments mapped.

Epicardial mapping was highly encouraged in cases where only partial diastolic pathway mapping was achieved endocardially. However, this was not uniformly performed. The breakdown of the partial map group is as follows: 6/24 patients had undergone previous cardiac surgery and were not candidates for epicardial access. Eleven out of 24 patients underwent epicardial mapping; however, recording of the diastolic pathway remained partial.

Of the remaining 7 partial map patients: 4 patients had 2/3 of the diastolic interval mapped, along with VT interruption during radiofrequency, in addition to VT noninducibility; epicardial access was not performed given the end points achieved. Two patients demonstrated VT circuits located at the interventricular septum, therefore, epicardial access was not performed; 1 patient developed progressive hemodynamic deterioration following endocardial mapping limiting epicardial access.

No recording of the diastolic pathway of the clinical VT was feasible in 25/85 patients (29.4%). Reasons for no diastolic recording included (1) progressive hemodynamic deterioration during mapping requiring pace termination or cardioversion following induction and mapping of the clinical VT (>30 seconds); (2) catheter induced termination with subsequent inability to reinduce the clinical VT despite repeated attempts at PVS following induction and mapping of the clinical VT (>30 seconds); (3) intramural substrate in cases where both endocardial and epicardial access were undertaken; (4) Epicardial substrate in cases where epicardial access was contraindicated.

Full recording of the diastolic pathway was more frequently achieved in ICM patients (26/36, 72%) as compared to patients with NICM (10/36, 28%). The difference between the 2 groups was statistically significant (*P*=0.004). Results of diastolic mapping according to etiology are reported in Table 2.

**Table 2.**
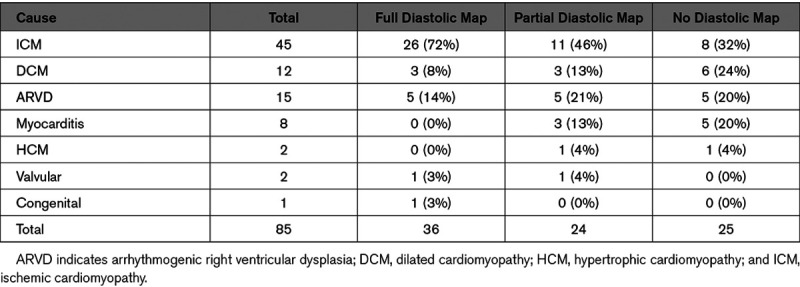
Diastolic Pathway Mapping by Cause

### Procedure End Points

#### VT Termination

The arrhythmia terminated during radiofrequency ablation in 22/36 (61%) patients in the full pathway group. VT termination during radiofrequency ablation occurred in 13/24 (54%) in the partial pathway group. The difference between the 2 groups was nonsignificant (*P*=0.79).

#### Elimination of Near Field Activity

Elimination of near field electrograms at sites of diastolic activity during VT was confirmed in 36/36 full pathway patients and 24/24 partial pathway patients.

#### Elimination of LPs

Elimination of LPs at sites of SR conduction slowing was confirmed in 23/25 (92%) patients in the substrate modification group.

#### End Procedure PVS

PVS at end of procedure was performed in 76/85 (89%) patients with VT noninducibility shown in 68/76 (89%) patients. In the full pathway group, 29/32 (91%) were noninducible. In the partial pathway group, 20/22 (91%) were noninducible. In the substrate modification group, 18/22 (82%) were noninducible.

#### Freedom From VT Recurrence

**Figure 5.**
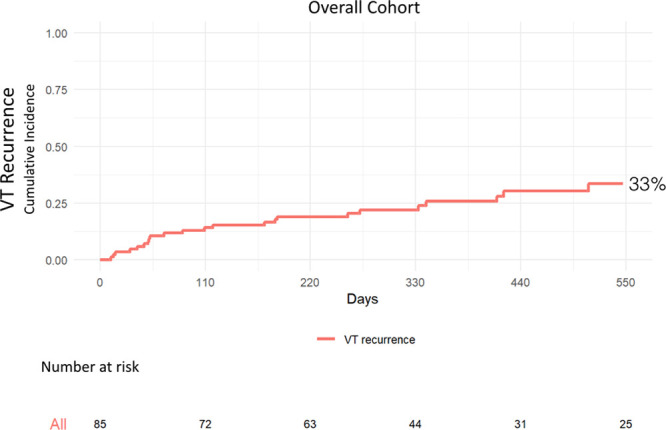
**Cumulative incidence of ventricular tachycardia (VT) recurrence of the overall cohort over 18 mo.**

**Figure 6.**
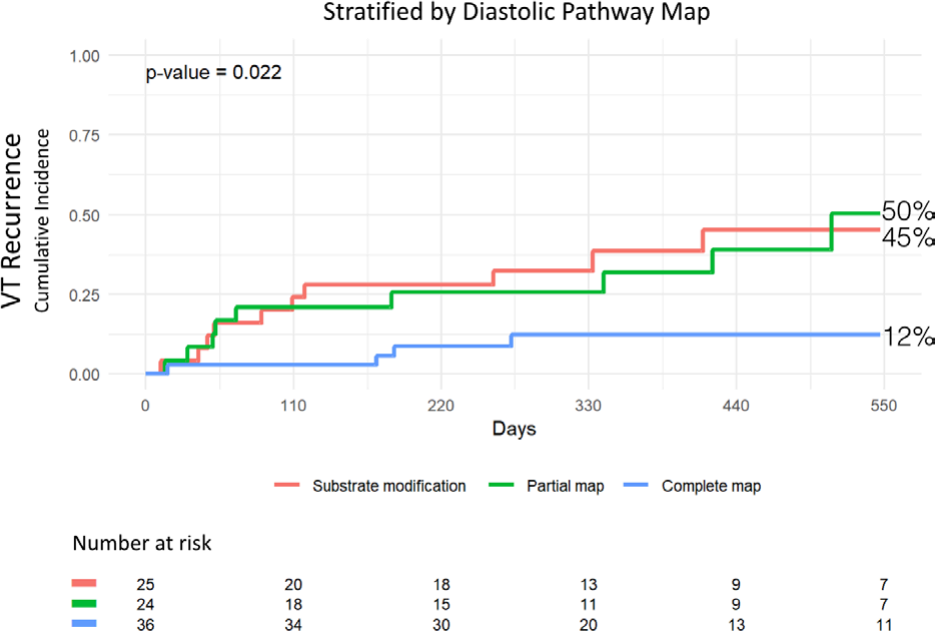
**Cumulative incidence of ventricular tachycardia (VT) recurrence stratified by diastolic pathway map over 18 mo.**

No patients were lost to follow-up. Mean follow-up was 12.8±5.2 months. At 18 months, the cumulative incidence of VT recurrence in the overall cohort was 33% representing a freedom from VT recurrence after the last ablation procedure of 67% (Figure 5). At 18 months, cumulative incidence of VT recurrence was 12%, representing a freedom from VT of 88%, in patients who had full diastolic activity recorded. Cumulative incidence of VT recurrence was 50%, representing a freedom from VT of 50%, in patients who had partial diastolic activity recorded. Cumulative incidence of VT recurrence was 45%, representing a freedom from VT of 55%, in patients who underwent substrate modification. The difference between the groups was statistically significant (*P*=0.02; Figure 6).

**Figure 7.**
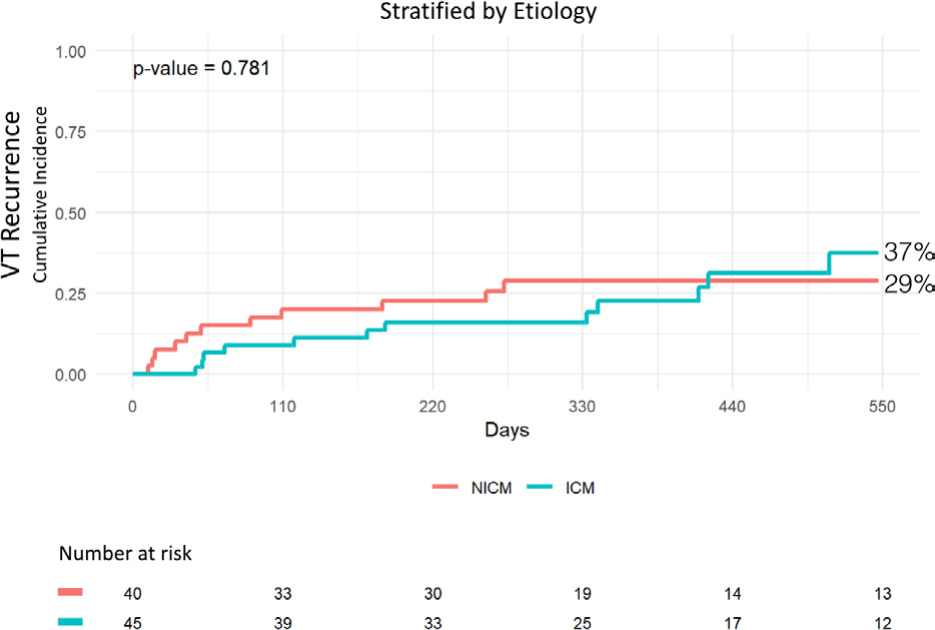
**Cumulative incidence of ventricular tachycardia (VT) recurrence in patients with ischemic cardiomyopathy (ICM) and nonischemic cardiomyopathy (NICM).**

A competing risk analysis stratified by cause (ICM versus NICM) was performed (Figure 7). At 18 months, cumulative incidence of VT recurrence was 37% and 29% in patients with ICM and NICM, respectively. The difference between the 2 groups was not statistically significant (*P*=0.78).

Univariable and multivariable Fine and Gray’s proportional subdistribution hazards regression models were built; the results are presented in Table 3. In multivariable analysis, the hazard ratios associated to the partial diastolic pathway group and full diastolic pathway group were, respectively, 0.81 (95% CI, 0.34–1.94, *P*=0.63) and 0.21 (95% CI, 0.07–0.63, *P*=0.005), while the number of induced VTs during the procedure had a hazard ratio of 1.52 (95% CI, 1.09–2.13, *P*=0.01) and amiodarone use post procedure had a hazard ratio of 2.39 (95% CI, 1.09–5.24, *P*=0.03).

**Table 3.**
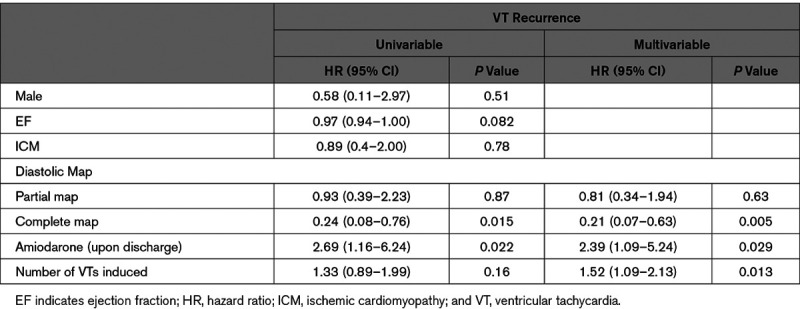
Univariable and Multivariable Competing Risk Regression Hazards Analysis of Baseline Covariates in Relation to Diastolic Pathway Map and Recurrence of VT

## Discussion

Key findings of the present study are the following: (1) mapping the full diastolic pathway was associated with higher freedom from VT recurrence as compared with those with partial or no mapping; and (2) full diastolic pathway recording of clinical VT can be achieved in 42% of patients with at least 30 seconds of mappable VT.

### Mapping Strategy

The benefits of catheter ablation of VT in patients with structural heart disease is increasingly established with freedom from VT recurrence approaching 70% at one year.^9^ Nonetheless, these success rates appear to have plateaued despite advances in mapping catheters as well as electroanatomic mapping systems. The finding of an 88% freedom from VT recurrence in patients with full diastolic pathway recording sheds light on the complexity of VT; in particular, the 3-dimensional nature of VT circuits.

### Full Versus Partial Diastolic Pathway Recording

Our approach to VT ablation emphasizes functional electrical imaging of the diastolic pathway. From initial placement of the catheter at sites of SR conduction slowing, then induction of VT with sequential movement of the catheter as guided by our distinct window of interest, focusing only from QRS offset to QRS onset, the goal is to accurately delineate the functional re-entrant pathway in a reasonable time frame. In doing so, the operator can appreciate the length, width, and extent of the reentry. As such, ablation in these patients targets all areas that are operational during VT. This, in part, may explain the demonstrably low rate of VT recurrence in the full map group, and the appreciable increase in recurrence rates in the partial map and substrate modification groups. Subsequent ablation in SR of all areas that had recorded diastolic activity may have guided us to a more complete ablation as compared with those without total mapping of the diastolic pathway where guidance was less precise. Notably, 3 cases of full diastolic pathway recording were achieved via combined endo-epicardial access. Given that combined endo-epicardial access was undertaken in only 4 of the 24 patients with partial diastolic pathway recording, one may surmise that combined mapping was underutilized in these patients. Epicardial access, if uniformly obtained as a first line approach in patients where detailed endocardial mapping fails to reveal the complete diastolic pathway, may add to the overall efficacy of the procedure. However, further interpretation of this discrepancy in recurrence rates may be explained by the 3-dimensional nature of VT.

### VT in 3-Dimensional

Diastolic intramural activation, where activation mapping was incomplete in endocardium and epicardium during VT, has been demonstrated using intraoperative panoramic simultaneous endo/epicardial mapping with an intramural multielectrode plunge needle for mapping.^10^ In this series, predominant intramural activation was observed in 40% of VTs with similar occurrence in both ICM and NICM. Our finding of a freedom from VT recurrence of 50% in those patients with only a partial diastolic pathway map appears consistent with this data. The inability to capture the entire diastolic pathway, despite adequate mapping time, suggests the presence of activation bridges in a 3-dimensional model of VT and the possibility of an intramural location of the re-entrant circuit. Patients with only a partial recording of the diastolic pathway may possess a greater amount of critical tissue residing intramurally where actual modification of the conduction properties is unfeasible.

Employing simultaneous endo-epicardial mapping, as recently described,^11,12^ may help address this issue by not only highlighting critical components of VT circuits that may extend from endo to epicardium, but by guiding ablation to the layer of interest.

### ICM and Full Diastolic Mapping: Addressing the Relationship

Our results show a significantly higher representation of patients with ICM in the full diastolic pathway group as compared with patients with NICM. Given that patients with ICM historically have higher VT ablation success rates compared with NICM,^9^ we sought to explore this potential confounding effect on the relationship of interest. To address this issue, we first estimated the cumulative incidence of VT recurrence stratified by cause (ICM versus NICM) which did not demonstrate a statistically significant difference (*P*=0.78). Furthermore, our multivariable analysis did not support cause as a significant predictor of VT recurrence, while type of diastolic map achieved retained a strong association. As such, we believe it reasonable that greater detailed VT circuit identification is indeed the reason from improved outcomes, rather than ischemic cause.

### Radiofrequency Termination and Negative PVS: Fallacious End Points?

Our results demonstrate similar rates of VT termination with radiofrequency between the full and partial diastolic pathway groups (61% and 52%, respectively) despite a stark contrast in VT recurrence rates at 18 months. While termination of VT with radiofrequency is accepted as proof of ablation of a site critical to the maintenance of reentry, these results highlight a potential fallacy of this end point. While satisfying to the operator, radiofrequency termination of VT may in fact be the result from thermal lesion formation that will subsequently recede in the following days. Furthermore, without guidance to the complete extent of the VT circuit, future VTs may recur using the same circuitry left untouched by ablation.

This is further reflected in the rate of noninducibility with PVS at end procedure between all 3 mapping groups. Acute noninducibility at study end has already been shown to underestimate ablation outcome,^13^ with up to 26% of patients becoming subsequently inducible with PVS at day 6.^8^ While these deficiencies may be explained by differences in autonomic tone and degree of sedation with anesthesia, the difference in recurrence rates in the partial and substrate modification groups may in fact be reflecting the inability to target all aspects of the reentrant circuit in addition to electrophysiological recovery of ablated tissue.

### Clinical Implications

In patients presenting for VT ablation, the ability to record the full diastolic pathway appears to portend a high freedom from VT recurrence. This group of patients may ideally be targeted for ablation and subsequently considered for more conservative medical management, free from continued antiarrhythmic treatment, following ablation. Conversely, in those patients in whom incomplete diastolic activity is tracked on the endocardial surface, epicardial mapping should be considered as a first line indication to identify the missing link to record the entire diastolic pathway. Similarly, in patients presenting with septal VT and incomplete diastolic mapping, the operator should consider simultaneously mapping of the right ventricular aspect of the septum in an effort to identify the missing segments or to prove the presence of an intramural component, all the while concurrently preparing for bipolar radiofrequency ablation.^14^

### Limitations

The authors recognize that this is a single center study involving a small number of patients. Entrainment data was not available in all cases, as concerns over terminating VT or altering VT morphology would pose limits on the extent of VT mappability. This limited circuit identification to activation mapping alone. Furthermore, analysis was performed based on clinical VT, and not all VTs induced at time of ablation. This approach was chosen since the primary end point of this study was clinical, VT recurrence, and not directed at individual VT characteristics. However, this shortcoming may be somewhat mitigated by the fact that multiple VTs frequently share the same VT isthmus in patients.^15^ The rate of epicardial access in our series was 27%. While this rate is comparable to contemporary VT series,^9^ given the nature of our study, a higher number of combined endo-epicardial access may have provided additional information regarding potential intramural substrate. The follow-up having been closed at the first symptomatic recurrence poses a boundary on the precision of our estimates of the treatment effect; more precise evaluations could be made following a longitudinal approach. Finally, our mapping is based on a specific technology and mapping catheter (GMC). This catheter was chosen as a result of our center’s experience, based on its pliability within cardiac chambers and efficiency at mapping diastolic activity. We do not possess head to head data with other mapping catheters.

### Conclusions

Complete electroanatomic imaging of the full diastolic pathway may be achieved in a fair proportion of cases and is associated with a higher freedom from VT recurrence as compared with partial diastolic pathway recording and substrate modification. The use of multielectrode mapping catheters in recording diastolic activity may help predict those VTs employing intramural circuits and further optimize ablation strategies.

## Sources of Funding

None.

## Disclosures

Dr Della Bella is a consultant for Abbott and Biosense and has received research grants from Abbott, Biosense, Biotronik, and Boston Scientific. Dr Frontera discloses consultant fees from Abbott Medical; Boston Scientific and Biosense Webster. Dr Bisceglia discloses consultant fees from Abbott Medical. The other authors report no conflicts. Open Access for this study has been made possible with the unconditional support of Abbott.

## Supplementary Material


