# Supplementary material for: Complete Electroanatomic Imaging of the Diastolic Pathway Is Associated With Improved Freedom From Ventricular Tachycardia Recurrence
Source: Circ Arrhythm Electrophysiol. 2020 Jul 28;13(9):e008651. doi: 10.1161/CIRCEP.120.008651 (PMC7495983; doi:10.1161/CIRCEP.120.008651)
Supplement: Supplementary file 1 [file hae-13-e008651-s001.pdf]

SUPPLEMENTAL MATERIAL

Supplemental video legend:

RF ablation demonstrating termination of VT upon completion of the ablation line transecting the isthmus.
