# Supplementary material for: Complete Electroanatomic Imaging of the Diastolic Pathway Is Associated With Improved Freedom From Ventricular Tachycardia Recurrence
Source: Circ Arrhythm Electrophysiol. 2020 Jul 28;13(9):e008651. doi: 10.1161/CIRCEP.120.008651 (PMC7495983; doi:10.1161/CIRCEP.120.008651)

Complete Electroanatomic Imaging of the Diastolic Pathway is Associated with Improved Freedom from Ventricular Tachycardia Recurrence

Running title: *Hadjis et al.; The Role of High-Density Mapping of VT*

Alexios Hadjis, MD[1]; Antonio Frontera, MD, PhD[1]; Luca Rosario Limite, MD[1]; Caterina Bisceglia, MD[1]; Ludovica Bognoni, MD[2]; Luca Foppoli, MSc[1]; Felicia Lipartiti, MD[1]; Gabriele Paglino, MD[1]; Andrea Radinovic, MD[1]; Giorgio Tsitsinakis, MD, PhD[1]; Federico Calore, MSc[3]; Paolo Della Bella, MD[1]

[1]Arrhythmology Department, [2]University of Medicine, IRCCS San Raffaele Hospital; [3]Abbott Medical Italy, Sesto San Giovanni, Milano, Italy

Circulation: Arrhythmia and Electrophysiology

Correspondence:
Alexios E. Hadjis, MD
IRCCS San Raffaele Hospital
Arrhythmology
Via Olgettina Milano, 60
Milano, 20132
Italy
Tel: +393498287364
Email: alexios.e.hadjis@gmail.com

Journal Subject Terms: Electrophysiology; Arrhythmias

Abstract:

Background - The development of multielectrode mapping catheters has expanded the spectrum of mappable ventricular tachycardias (VTs). Full diastolic pathway recording has been associated with a high rate of VT termination during radiofrequency (RF) ablation as well as non inducibility at study end. However, the role of diastolic pathway mapping on VT recurrence has yet to be clearly elucidated. We aimed to explore the role of complete diastolic pathway activation mapping on VT recurrence.

Methods - 85 consecutive patients who underwent VT ablation guided by high-density mapping were enrolled. During activation mapping, the presence of electrical activity in all segments of diastole defined the evidence of having had recorded the whole diastolic interval. Patients were categorized as having recorded the full diastolic pathway, partial diastolic pathway, or no diastolic pathway map performed. Recurrences of VT were defined as appropriate ICD therapies or on the basis of ECG-documented arrhythmia.

Results - 85 patients were included. Complete recording of the diastolic pathway was achieved in 36/85 (42.4%) patients. Partial recording of the diastolic pathway of the clinical VT was achieved in 24/85 (28.2%) patients. No recording of the diastolic pathway of the clinical VT was feasible in 25/85 patients (29.4%). At a mean of 12.8 months, freedom from VT recurrence was 67% in the overall cohort. At a mean of 12.8 months, freedom from VT recurrence was 88%, 50%, and 55% in patients who had full diastolic activity recorded, partial diastolic activity recorded, or underwent substrate modification, respectively; the observed differences were statistically significant (p=0.02).

Conclusions - Mapping of the entire diastolic pathway was associated with a higher freedom from VT recurrence as compared to partial diastolic pathway recording and substrate modification. The use of multielectrode mapping catheters in recording diastolic activity may help predict those VTs employing intramural circuits and further optimize ablation strategies.

Key words: ventricular arrhythmia; mapping; ablation; electrophysiology mapping

Circulation: Arrhythmia and Electrophysiology

Nonstandard Abbreviations and Acronyms

ATP – Anti Tachycardia Pacing

CRT – Cardiac Resynchronization Therapy

ECG – Electrocardiogram

GMC – Grid Mapping Catheter

ICD – Implantable Cardioverter Defibrillator

ICM – Ischemic Cardiomyopathy

LP – Late Potential

LVEF – Left Ventricular Ejection Fraction

NICM – Non Ischemic Cardiomyopathy

PVS – Programmed Ventricular Stimulation

RF - Radiofrequency

TCL – Tachycardia Cycle Length

VT – Ventricular Tachycardia

American Heart Association.
Circulation: Arrhythmia and Electrophysiology

Introduction

Catheter ablation of ventricular tachycardia (VT) is increasingly performed worldwide[1,2], however, success rates of ablation have plateaued[3]. While numerous approaches to ablation have been developed, particularly in the domain of substrate modification[4], the advancements in multi-electrode mapping catheters have expanded the spectrum of mappable VTs. The increased number of electrodes, in addition to extent of the recording area and electrode orientation, have proven useful in creating fast and reliable activation maps during VT. Most importantly, in cases of hemodynamically unstable VT, these maps can be produced in a reasonable time frame[5] . Full diastolic pathway recording has been associated with a high rate of VT termination during RF ablation as well as non inducibility at study end[5]. However, the role of diastolic pathway mapping on VT recurrence has yet to be clearly elucidated. We sought to explore how

frequently full electroanatomic imaging of the diastolic pathway mapping can be achieved and how this translates to clinical outcome.

Methods

The data that support the findings of this study are available from the corresponding author upon reasonable request.

Study Population

A retrospective analysis was performed in patients referred to our institution for VT ablation from December 2017 to June 2019 whom underwent ablation with the EnSite Precision™ Cardiac Mapping System (Abbott, MN, USA), guided by high-density mapping with the Grid mapping catheter (GMC) (Advisor HD Grid Mapping Catheter Sensor Enabled™, Abbott, MN, USA). Patients with both ischemic cardiomyopathy (ICM) and non ischemic cardiomyopathy (NICM) were included in the study. Only patients with at least 30 seconds of mappable VT were included. Patients with a recent acute coronary syndrome (ACS), recent coronary artery bypass grafting (CABG), or any history of left ventricular assist device (LVAD) implant were excluded. The study was approved by the institutional review committee, and all patients gave their written informed consent.

American Heart Association.
Circulation: Arrhythmia and Electrophysiology

Definitions

VTs induced at the time of ablation were defined as clinical if they matched the arrhythmia captured clinically on 12 lead electrocardiogram (ECG) or, in cases where no ECG was available, they matched the tachycardia cycle length (TCL) of the implantable cardioverter defibrillator (ICD) log.

Arrhythmia recurrence was defined as any arrhythmia receiving device-based treatments [anti tachycardia pacing (ATP) or shock] or any VT episodes assessed at clinical evaluation.

Workflow

The procedures were performed under general anesthesia, with continuous invasive monitoring of the arterial pressure, arterial oxygen saturation, and acid/base balance, according to our standard protocol as previously described[6]. In cases where no 12 lead ECG of the clinical VT was available, PVS with a standard fixed curve quadripolar catheter at the right ventricular apex was performed at study onset under conscious sedation prior to induction of general anesthesia.

Epicardial mapping and ablation was favoured in patients with a history of previously failed endocardial ablation, myocarditis, or arrhythmogenic right ventricular dysplasia (ARVD). Epicardial access, in cases where a detailed endocardial mapping during VT failed to reveal the complete diastolic interval, was attempted within the same procedure unless considered inadvisable due to hemodynamic instability or overall unfavourable patient conditions. Epicardial access was not attempted in patients with a previous history of cardiac surgery.

American Heart Association.
Circulation: Arrhythmia and Electrophysiology

At first, a geometry of the chamber of interest was created with a Flexability Ablation Catheter Sensor Enabled ™ (Abbott, MN, USA); then, a GMC was used to generate the substrate map either during sinus rhythm or right ventricular pacing in pacing dependent patients. In those patients with cardiac resynchronization therapy (CRT) devices, left ventricular pacing was turned off. Voltage map data were achieved using the GMC in the HD Wave bipolar configuration and the Best Duplicate Algorithm as previously described[5]. Isochronal late activation mapping, where SR propagation was displayed with eight equally distributed isochrones of activation, was routinely performed to investigate the presence isochronal crowding (>2 isochrones within a 1

cm radius) and conduction delay of propagation. SR activation map timing was set at the termination of the latest EGM deflection at any given point. Subsequently, PVS (up to 4 extrastimuli delivered at the right ventricular apex and multiple left ventricular sites) was performed to induce ventricular tachycardia.

Diastolic pathway mapping

Activation mapping was attempted on all induced arrhythmias by sequentially positioning the GMC, starting at sites showing the maximum conduction delay at baseline sinus rhythm (SR) mapping. This was guided by visual identification of conduction slowing, based on isochronal late activation mapping, as previously described[7], performed during SR. The window of interest was then opened from the termination of the first QRS to the onset of the second QRS of the VT cycle, to define the diastolic interval. The diastolic interval was then divided into 3 main segments, as defined by the timing of the first deflection of the electrogram recorded at any bipole during the VT diastolic interval:

1. 0-35% of the diastolic interval defined entrance
2. 36-65% of the diastolic interval defined isthmus
3. 66-100% of the diastolic interval defined exit

According to the extent of the electrogram recording during mapping, patients were categorized as either having recorded the full diastolic pathway, partial diastolic pathway, or no diastolic pathway of the clinical VT.

Full diastolic pathway mapping was defined as having recorded the presence of electrical activity in all segments, bridging each QRS to the next one (Figure 1).

Partial diastolic pathway mapping was defined as having recorded electrical activity in at least 1 of the 3 diastolic segments.

Circulation: Arrhythmia and Electrophysiology
American Heart Association.

No diastolic pathway mapping indicated that no EGMs were seen in the diastolic window despite detailed mapping of the VT and were defined as the SR substrate modification group.

Ablation

In cases where electrical activity was present throughout the entire diastolic interval, a fully functional imaging of the diastolic pathway was displayed. Utilizing this functional electrical imaging of the re-entry, demonstrating both extent and width of the channel, in addition to funnels at the entry and exit, the ablation strategy targeted interruption of the reentrant activation at the narrowest point between the boundaries of the VT channel (supplementary video). A line of ablation was performed at the narrowest site, usually between entrance and isthmus sites.

This was performed during VT, or in cases of hemodynamically non tolerated VT, following arrhythmia termination during sinus or paced rhythm by delivering contiguous lesions to organize a transecting line at the described level using the VT activation map as reference. Complete elimination of near field activity at sites involving the VT re-entry was confirmed with subsequent remap confirming elimination of near field activity during SR (Figure 2).

American Heart Association.
Circulation: Arrhythmia and Electrophysiology

In cases where the diastolic pathway map was not completed, zones of isochronal crowding, in addition to zones of conduction slowing, visually confirmed with SR LAT propagation maps, were targeted for ablation until LPs were modified or eliminated. LP abolition was routinely confirmed with re-mapping post ablation (Figure 3).

The power was set to 50 W, the tip temperature read <45°C, the irrigation rate was 17 mL/minute, the max impedance drop was 20 Ohms. Ablation lesions were performed for a duration of 60-120 seconds with catheter maneuvers performed to maintain catheter stability as guided by dedicated software tools (Automark) of the Ensite Precision™ Cardiac Mapping System.

Endpoints included all of the following: 1) VT termination; 2) Elimination of near field activity (ablation continued until absence of near field activity was confirmed at the sites where diastolic activity had been recorded from subsequent remaps performed with the GMC); 3) Elimination of LPs at sites of conduction slowing during SR; 4) VT non inducibility using the full induction protocol used at baseline (Figure 4).

Follow up

All patients were followed at 3-month intervals with remote monitoring or office visits where implantable devices were interrogated and during any symptomatic event. Two experienced electrophysiologists (A.F., A.H.) reviewed the stored ICD electrograms and adjudicated the arrhythmic events. Antiarrhythmic medications were discontinued upon hospital discharge if complete non inducibility was achieved. ICD programming, utilizing high rate ICD therapy cut-off criteria and delayed arrhythmia detection, was performed according to standard protocol[8].

American Heart Association.
Circulation: Arrhythmia and Electrophysiology

Statistical Analysis

The continuous variables are presented as mean ± standard deviation (SD) (if normally distributed) and median (interquartile range) otherwise; the categorical variables are reported as count (percentage).

To account for the effect of competing risks (i.e. death from all causes) on the estimates on the incidences relative to the event of interest (VT recurrences), cumulative incidence functions were computed; Gray's test was used for group comparison.. A multivariable Fine and Gray's proportional subdistribution hazards regression model was built to assess the relationship between the treatment (diastolic mapping – "substrate mapping" was used as reference category), the potential confounders (gender, age, ejection fraction, ischemic yes/no, amiodarone at discharge, number of VTs induced during the procedure - selected via backward selection) and

the event of interest (VT recurrence) over time. All tests were two-sided and p-values below 0.05 were considered statistically significant. R, version 3.6.2, was used for the analyses (packages: tidyverse, survival, survminer, pec, cmprsk, crrstep).

Results

Study Population

A total of 85 consecutive patients were enrolled in this study (mean age of 62.0±12.4 years, mean LVEF of 38.8 ±12.2%, mean LVEF excluding ARVD and myocarditis patients of 35.1+/-10.2) with a total of 147 VTs induced (mean VT CL 373 ± 94ms). The clinical characteristics of the study population are reported in Table 1. Epicardial access was obtained in 23 (27%) patients. Periprocedural complications included 3 cases of pericarditis, 2 femoral pseudoaneurysms, and 2 cases of cardiac tamponade treated successfully with pericardial drain. In the overall cohort, 18 (21%) of patients were discharged from hospital on amiodarone. Overall mortality was 7% at 18 months.

American Heart Association.
Circulation: Arrhythmia and Electrophysiology

Recording of the Diastolic Pathway.

Complete recording of the diastolic pathway of the clinical VT was achieved in 36/85 (42%) patients. The full diastolic pathway of the clinical VT was recorded endocardially only in 28/36 patients (78%), epicardially only in 5/36 (14%) patients, and endoepicardially combined access in 3/36 (8%).

Partial recording of the diastolic pathway of the clinical VT was achieved in 24/85 (28.2%) patients. The missing aspect of the diastolic pathway of the clinical VT occurred at the entry site in 18 patients, at the isthmus in 14 patients, and at the exit area in 7 patients. Overall, 6/24 (25%) patients had 2 of 3 segments mapped, and 18/24 (75%) had 1 of 3 segments mapped.

Epicardial mapping was highly encouraged in cases where only partial diastolic pathway mapping was achieved endocardially. However, this was not uniformly performed. The breakdown of the partial map group is as follows: 6/24 patients had undergone previous cardiac surgery and were not candidates for epicardial access. 11/24 patients underwent epicardial mapping; however, recording of the diastolic pathway remained partial.

Of the remaining 7 partial map patients: 4 patients had 2/3 of the diastolic interval mapped, along with VT interruption during RF, in addition to VT non inducibility; Epicardial access was not performed given the end points achieved. 2 patients demonstrated VT circuits located at the interventricular septum therefore epicardial access was not performed; 1 patient developed progressive hemodynamic deterioration following endocardial mapping limiting epicardial access.

American Heart Association.
Circulation: Arrhythmia and Electrophysiology

No recording of the diastolic pathway of the clinical VT was feasible in 25/85 patients (29.4%). Reasons for no diastolic recording included: 1) Progressive hemodynamic deterioration during mapping requiring pace termination or cardioversion following induction and mapping of the clinical VT (>30 seconds); 2) Catheter induced termination with subsequent inability to reinduce the clinical VT despite repeated attempts at PVS following induction and mapping of the clinical VT (>30 seconds); 3) Intramural substrate in cases where both endocardial and epicardial access were undertaken; 4) Epicardial substrate in cases where epicardial access was contraindicated.

Full recording of the diastolic pathway was more frequently achieved in ICM patients (26/36, 72%) as compared to NICM patients (10/36, 28%). The difference between the two groups was statistically significant ($p=0.004$). Results of diastolic mapping according to etiology are reported in Table 2.

Procedure End Points

VT Termination

The arrhythmia terminated during radiofrequency ablation in 22/36 (61%) patients in the full pathway group. VT termination during radiofrequency ablation occurred in 13/24 (54%) in the partial pathway group. The difference between the two groups was non-significant (p=0.79).

Elimination of near field activity

Elimination of near field EGMs at sites of diastolic activity during VT was confirmed in 36/36 full pathway patients and 24/24 partial pathway patients.

Elimination of LPs.

Elimination of LPs at sites of SR conduction slowing was confirmed in 23/25 (92%) patients in the substrate modification group.

American Heart Association.

End Procedure PVS

Circulation: Arrhythmia and Electrophysiology

PVS at end of procedure was performed in 76/85 (89%) patients with VT noninducibility shown in 68/76 (89%) patients. In the full pathway group, 29/32 (91%) were non inducible. In the partial pathway group, 20/22 (91%) were non inducible. In the substrate modification group, 18/22 (82%) were non inducible.

Freedom from VT recurrence

No patients were lost to follow up. Mean follow up was 12.8 ± 5.2 months. At 18 months, the cumulative incidence of VT recurrence in the overall cohort was 33% representing a freedom from VT recurrence after the last ablation procedure of 67% (Figure 5). At 18 months, cumulative incidence of VT recurrence was 12%, representing a freedom from VT of 88%, in patients who had full diastolic activity recorded. Cumulative incidence of VT recurrence was 50%, representing a freedom from VT of 50%, in patients who had partial diastolic activity

recorded. Cumulative incidence of VT recurrence was 45%, representing a freedom from VT of 55%, in patients who underwent substrate modification. The difference between the groups was statistically significant (p=0.02) (Figure 6).

A competing risk analysis stratified by etiology (ICM vs NICM) was performed (Figure 7). At 18 months, cumulative incidence of VT recurrence was 37% and 29% in ICM patients and NICM patients, respectively. The difference between the two groups was not statistically significant (p=0.78).

Univariable and multivariable Fine and Gray's proportional subdistribution hazards regression models were built; the results are presented in Table 3. In multivariable analysis, the HRs associated to the partial diastolic pathway group and full diastolic pathway group were, respectively, 0.81 (95% CI 0.34-1.94, p=0.63) and 0.21 (95% CI 0.07-0.63, p=0.005), while the number of induced VTs during the procedure had a HR of 1.52 (95% CI 1.09-2.13, p=0.01) and amiodarone use post procedure had a HR of 2.39 (95% CI 1.09-5.24, p=0.03).

American Heart Association.
Circulation: Arrhythmia and Electrophysiology

Discussion

Key findings of the present study are the following: 1) mapping the full diastolic pathway was associated with higher freedom from VT recurrence as compared to those with partial or no mapping. 2) full diastolic pathway recording of clinical VT can be achieved in 42% of patients with at least 30 seconds of mappable VT.

Mapping strategy

The benefits of catheter ablation of VT in patients with structural heart disease is increasingly established with freedom from VT recurrence approaching 70% at one year[9]. Nonetheless, these success rates appear to have plateaued despite advances in mapping catheters as well as

electroanatomic mapping systems. The finding of an 88% freedom from VT recurrence in patients with full diastolic pathway recording sheds light on the complexity of VT; in particular, the three-dimensional nature of VT circuits.

Full vs Partial Diastolic Pathway Recording

Our approach to VT ablation emphasizes functional electrical imaging of the diastolic pathway. From initial placement of the catheter at sites of SR conduction slowing, then induction of VT with sequential movement of the catheter as guided by our distinct window of interest, focusing only from QRS offset to QRS onset, the goal is to accurately delineate the functional re-entrant pathway in a reasonable time frame. In doing so, the operator can appreciate the length, width, and extent of the re-entry. As such, ablation in these patients targets all areas that are operational during VT. This, in part, may explain the demonstrably low rate of VT recurrence in the full map group, and the appreciable increase in recurrence rates in the partial map and substrate modification groups. Subsequent ablation in SR of all areas that had recorded diastolic activity may have guided us to a more complete ablation as compared to those without total mapping of the diastolic pathway where guidance was less precise. Notably, 3 cases of full diastolic pathway recording were achieved via combined endo-epicardial access. Given that combined endo-epicardial access was undertaken in only 4 of the 24 patients with partial diastolic pathway recording, one may surmise that combined mapping was underutilized in these patients. Epicardial access, if uniformly obtained as a first line approach in patients where detailed endocardial mapping fails to reveal the complete diastolic pathway, may add to the overall efficacy of the procedure. However, further interpretation of this discrepancy in recurrence rates may be explained by the three-dimensional nature of VT.

American Heart Association.
Circulation: Arrhythmia and Electrophysiology

VT in 3 dimensions

Diastolic intramural activation, where activation mapping was incomplete in endocardium and epicardium during VT, has been demonstrated utilizing intraoperative panoramic simultaneous endo/epicardial mapping with an intramural multi-electrode plunge needle for mapping[10]. In this series, predominant intramural activation was observed in 40% of VTs with similar occurrence in both ICM and NICM. Our finding of a freedom from VT recurrence of 50% in those patients with only a partial diastolic pathway map appears consistent with this data. The inability to capture the entire diastolic pathway, despite adequate mapping time, suggests the presence of activation bridges in a 3D model of VT and the possibility of an intramural location of the re-entrant circuit. Patients with only a partial recording of the diastolic pathway may possess a greater amount of critical tissue residing intramurally where actual modification of the conduction properties is unfeasible.

American Heart Association.
Circulation: Arrhythmia and Electrophysiology

Employing simultaneous endo-epicardial mapping, as recently described[11,12], may help address this issue by not only highlighting critical components of VT circuits that may extend from endo to epicardium, but by guiding ablation to the layer of interest.

ICM and full diastolic mapping: Addressing the relationship.

Our results show a significantly higher representation of ICM patients in the full diastolic pathway group as compared to NICM patients. Given that patients with ICM historically have higher VT ablation success rates compared to NICM[9], we sought to explore this potential confounding effect on the relationship of interest. In order to address this issue, we first estimated the cumulative incidence of VT recurrence stratified by etiology (ICM vs NICM) which did not demonstrate a statistically significant difference (p=0.78). Furthermore, our multivariable analysis did not support etiology as a significant predictor of VT recurrence, while

type of diastolic map achieved retained a strong association. As such, we believe it reasonable that greater detailed VT circuit identification is indeed the reason from improved outcomes, rather than ischemic etiology.

RF Termination and negative PVS: Fallacious end points?

Our results demonstrate similar rates of VT termination with RF between the full and partial diastolic pathway groups (61% and 52%, respectively) despite a stark contrast in VT recurrence rates at 18 months. While termination of VT with RF is accepted as proof of ablation of a site critical to the maintenance of reentry, these results highlight a potential fallacy of this end point. While satisfying to the operator, RF termination of VT may in fact be the result from thermal lesion formation that will subsequently recede in the following days. Furthermore, without guidance to the complete extent of the VT circuit, future VTs may recur utilizing the same circuitry left untouched by ablation.

Circulation: Arrhythmia and Electrophysiology

This is further reflected in the rate of non inducibility with PVS at end procedure between all 3 mapping groups. Acute non inducibility at study end has already been shown to underestimate ablation outcome[13], with up to 26% of patients becoming subsequently inducible with PVS at day 6[8]. While these deficiencies may be explained by differences in autonomic tone and degree of sedation with anesthesia, the difference in recurrence rates in the partial and substrate modification groups may in fact be reflecting the inability to target all aspects of the reentrant circuit in addition to electrophysiological recovery of ablated tissue.

Clinical Implications

In patients presenting for VT ablation, the ability to record the full diastolic pathway appears to portend a high freedom from VT recurrence. This group of patients may ideally be targeted for ablation and subsequently considered for more conservative medical management, free from

continued antiarrhythmic treatment, following ablation. Conversely, in those patients in whom incomplete diastolic activity is tracked on the endocardial surface, epicardial mapping should be considered as a first line indication in order to identify the missing link to record the entire diastolic pathway. Similarly, in patients presenting with septal VT and incomplete diastolic mapping, the operator should consider simultaneously mapping of the right ventricular aspect of the septum in an effort to identify the missing segments or to prove the presence of an intramural component, all the while concurrently preparing for bipolar RF ablation[14].

Limitations

The authors recognize that this is a single center study involving a small number of patients. Entrainment data was not available in all cases, as concerns over terminating VT or altering VT morphology would pose limits on the extent of VT mappability. This limited circuit identification to activation mapping alone. Furthermore, analysis was performed based on clinical VT, and not all VTs induced at time of ablation. This approach was chosen since the primary endpoint of this study was clinical, VT recurrence, and not directed at individual VT characteristics. However, this shortcoming may be somewhat mitigated by the fact that multiple VTs frequently share the same VT isthmus in patients[15] . The rate of epicardial access in our series was 27%. While this rate is comparable to contemporary VT series[9], given the nature of our study, a higher number of combined endo-epicardial access may have provided additional information regarding potential intramural substrate. The follow-up having been closed at the first symptomatic recurrence poses a boundary on the precision of our estimates of the treatment effect; more precise evaluations could be made following a longitudinal approach. Finally, our mapping is based on a specific technology and mapping catheter (GMC). This catheter was chosen as a result of our center's experience, based on its pliability within cardiac chambers and

American Heart Association.
Circulation: Arrhythmia and Electrophysiology

efficiency at mapping diastolic activity. We do not possess head to head data with other mapping catheters.

Conclusions

Complete electroanatomic imaging of the full diastolic pathway may be achieved in a fair proportion of cases and is associated with a higher freedom from VT recurrence as compared to partial diastolic pathway recording and substrate modification. The use of multielectrode mapping catheters in recording diastolic activity may help predict those VTs employing intramural circuits and further optimize ablation strategies.

Sources of Funding: None.

American Heart Association.

Disclosures: Dr Della Bella is a consultant for Abbott and Biosense and has received re- search grants from Abbott, Biosense, Biotronik, and Boston Scientific. Dr Frontera discloses consultant fees from Abbott Medical; Boston Scientific and Biosense Webster. Dr Bisceglia discloses consultant fees from Abbott Medical. The other authors report no conflicts.

Circulation: Arrhythmia and Electrophysiology

References:

1. Della Bella P, Baratto F, Tsiachris D, Trevisi N, Vergara P, Bisceglia C, Petracca F, Carbucicchio C, Benussi S, Maisano F, et al. Management of ventricular tachycardia in the setting of a dedicated unit for the treatment of complex ventricular arrhythmias: long-term outcome after ablation. *Circulation*. 2013;127:1359–68.

2. Sapp JL, Wells GA, Parkash R, Stevenson WG, Blier L, Sarrazin J-F, Thibault B, Rivard L, Gula L, Leong-Sit P, et al. Ventricular Tachycardia Ablation versus Escalation of Antiarrhythmic Drugs. *N Engl J Med*. 2016;375:111–21.

3. Liang JJ, Santangeli P, Callans DJ. Long-term Outcomes of Ventricular Tachycardia Ablation in Different Types of Structural Heart Disease. *Arrhythm Electrophysiol Rev.* 2015;4:177-183

4. Di Biase L, Burkhardt JD, Lakkireddy D, Carbucicchio C, Mohanty S, Mohanty P, Trivedi C, Santangeli P, Bai R, Forleo G, et al. Ablation of Stable VTs Versus Substrate Ablation in Ischemic Cardiomyopathy. *J Am Coll Cardiol*. 2015;66:2872–82.

5. Okubo K, Frontera A, Bisceglia C, Paglino G, Radinovic A, Foppoli L, Calore F, Della Bella P. Grid Mapping Catheter for Ventricular Tachycardia Ablation. *Circ Arrhythm Electrophysiol*. 2019;12: e007500.

6. Silberbauer J, Oloriz T, Maccabelli G, Tsiachris D, Baratto F, Vergara P, Mizuno H, Bisceglia C, Marzi A, Sora N, et al. Noninducibility and late potential abolition: a novel combined prognostic procedural end point for catheter ablation of postinfarction ventricular tachycardia. *Circ Arrhythm Electrophysiol*. 2014;7:424–35.

7. Irie T, Yu R, Bradfield JS, Vaseghi M, Buch EF, Ajijola O, Macias C, Fujimara O, Mandapati R, Boyle NG, et al. Relationship Between Sinus Rhythm Late Activation Zones and Critical Sites for Scar-Related Ventricular Tachycardia: Systematic Analysis of Isochronal Late Activation Mapping. *Circ Arrhythm Electrophysiol*. 2015;8:390–9.

8. Oloriz T, Baratto F, Trevisi N, Barbaro M, Bisceglia C, D'Angelo G, Yamase M, Paglino G, Radinovic A, Della Bella P. Defining the Outcome of Ventricular Tachycardia Ablation: Timing and Value of Programmed Ventricular Stimulation. *Circ Arrhythm Electrophysiol*. 2018;11:e005602.

9. Tung R, Vaseghi M, Frankel DS, Vergara P, Di Biase L, Nagashima K, Yu R, Vangala S, Tseng CH, Choi EK, et al. Freedom from recurrent ventricular tachycardia after catheter ablation is associated with improved survival in patients with structural heart disease: An International VT Ablation Center Collaborative Group study. *Heart Rhythm*. 2015;12:1997–2007.

10. Bhaskaran A, Nayyar S, Porta-Sánchez A, Jons C, Massé S, Magtibay K, Aukhojee P, Ha A, Bokhari M, Tung R, et al. Direct and indirect mapping of intramural space in ventricular tachycardia. Heart Rhythm. 2020;17:439–46.

11. Frontera A, Hadjis A, Calore F, Della Bella P. Simultaneous endo-epicardial-high density mapping of ventricular tachycardia with the use of multi-electrode mapping catheters. *J Interv Card Electrophysiol*. 2020; https://doi.org/10.1007/s10840-020-00762-6

12. Tung R, Raiman M, Liao H, Zhan X, Chung FP, Nagel R, Hu H, Jian J, Shatz DY, Besser SA, et al. Simultaneous Endocardial and Epicardial Delineation of 3D Reentrant Ventricular Tachycardia. *J Am Coll Cardiol*. 2020;75:884–97.

13. de Riva M, Piers SRD, Kapel GFL, Watanabe M, Venlet J, Trines SA, Schalij MJ, Zeppenfeld K. Reassessing noninducibility as ablation endpoint of post-infarction ventricular tachycardia: the impact of left ventricular function. *Circ Arrhythm Electrophysiol*. 2015;8:853–62.

14. Sauer WH, Steckman DA, Zipse MM, Tzou WS, Aleong RG. High-power bipolar ablation for incessant ventricular tachycardia utilizing a deep midmyocardial septal circuit. *HeartRhythm Case Rep*. 2015;1:397–400.

15. Martin R, Hocini M, Haïsaguerre M, Jaïs P, Sacher F. Ventricular Tachycardia Isthmus Characteristics: Insights from High-density Mapping. *Arrhythmia Electrophysiol Rev*. 2019;8:54.

American Heart Association.
Circulation: Arrhythmia and Electrophysiology

Table 1. Patient Characteristics and Procedure Details

Patients	N=85
Age (years)	62 ± 12
Sex (M)	79 (93%)
Etiology	
ICM	45 (53%)
DCM	12 (14%)
ARVD	15 (18%)
Valvular	2 (2%)
Myocarditis	8 (9%)
Congenital	1 (1%)
HCM	2 (2%)
LVEF	38 ± 12
LVEF (%, excluding ARVD, myocarditis)	35 ± 10
Procedure (1st/2nd/≥3rd)	57/19/9
Amiodarone on admission	36 (42%)
≥ 2 antiarrhythmic drugs	6 (7%)
Amiodarone on discharge	18 (21%)
Prior cardiac surgery	28 (33%)
**Access**	
Endo	62 (73%)
Epi	11 (13%)
Endo + Epi	12 (14%)
**VTs overall**	147
**End Points**	
PES at study end	76 (89%)
VT non inducible	68 (89%)

Unless otherwise indicated, the continuous variables are presented as mean ± SD and the categorical ones as count (percentage).
LVEF: left ventricular ejection fraction, ICM: ischemic cardiomyopathy, DCM: dilated cardiomyopathy; ARVD: arrhythmogenic right ventricular dysplasia; HCM: hypertrophic cardiomyopathy; VT: ventricular tachycardia; Endo: endocardial; Epi: Epicardial; PES: Programmed Extrastimulation.

American Heart Association.
Circulation: Arrhythmia and Electrophysiology

Table 2. Diastolic Pathway Mapping by Etiology

Etiology	Total	Full Diastolic Map	Partial Diastolic Map	No Diastolic Map
ICM	45	26 (72%)	11 (46%)	8 (32%)
DCM	12	3 (8%)	3 (13%)	6 (24%)
ARVD	15	5 (14%)	5 (21%)	5 (20%)
Myocarditis	8	0 (0%)	3 (13%)	5 (20%)
HCM	2	0 (0%)	1 (4%)	1 (4%)
Valvular	2	1 (3%)	1 (4%)	0 (0%)
Congenital	1	1 (3%)	0 (0%)	0 (0%)
Total	85	36	24	25

ICM: ischemic cardiomyopathy, DCM: dilated cardiomyopathy; ARVD: arrhythmogenic right ventricular dysplasia; HCM: hypertrophic cardiomyopathy

American Heart Association.
Circulation: Arrhythmia and Electrophysiology

Table 3. Univariable and Multivariable Competing Risk Regression Hazards Analysis of Baseline Covariates in Relation to Diastolic Pathway Map and Recurrence of VT.

	**VT Recurrence**			
	**Univariable**		**Multivariable**	
	HR (95% CI)	*P* Value	HR (95% CI)	*P* Value
Male	0.58 (0.11-2.97)	0.51		
EF	0.97 (0.94-1.00)	0.082		
ICM	0.89 (0.4-2.00)	0.78		
**Diastolic Map**				
Partial map	0.93 (0.39-2.23)	0.87	0.81 (0.34-1.94)	0.63
Complete map	0.24 (0.08-0.76)	0.015	0.21 (0.07-0.63)	0.005
Amiodarone (upon discharge)	2.69 (1.16-6.24)	0.022	2.39 (1.09-5.24)	0.029
Number of VTs induced	1.33 (0.89-1.99)	0.16	1.52 (1.09-2.13)	0.013

ICM: ischemic cardiomyopathy, EF: ejection fraction; VT: ventricular tachycardia; CI: confidence interval; HR: hazard ratio.

American Heart Association.

Circulation: Arrhythmia and Electrophysiology

Figure Legends:

Figure 1. Ventricular tachycardia (VT) with the full diastolic pathway identified on epicardium with respective EGMs. As the window of interest is set at the diastolic phase, the diastolic pathway is demonstrated with sequential mappings from entry, through the isthmus, and to exit. First deflection annotation timing is marked by yellow bars for the respective electrograms.

Figure 2. Ablation strategy. (A) VT activation map displaying mid-isthmus to exit activity, encircled in red. (B) Ablation lesion set targeting diastolic activity. (C) Near field EGMs during

SR mapping at same site of VT reentry prior to ablation. (D) Remap illustrates the end point of near field activity abolition following ablation at the site of diastolic activity.

Figure 3. Substrate modification ablation strategy. (A) ILAM reconstruction of SR activation demonstrating isochronal crowding along the inferobasal LV. (B) SR activation map demonstrating late potentials at the same site using last deflection detection timing. (C) Following catheter ablation at site of conduction slowing, remap shows abolition of late potentials.

Figure 4. Ablation end points. (A) Termination of VT within 3.1 seconds of RF and associated catheter position (B) at mid isthmus. (C) Pre and post ablation SR activation maps demonstrating abolition of LPs at site of SR conduction slowing. (D) Post ablation, full PVS protocol is performed with VT non inducibility demonstrated.

American Heart Association.
Circulation: Arrhythmia and Electrophysiology

Figure 5. Cumulative incidence of VT recurrence of the overall cohort over 18 months

Figure 6. Cumulative incidence of VT recurrence stratified by diastolic pathway map over 18 months

Figure 7. Cumulative incidence of VT recurrence in ICM and NICM patients

What Is Known?

- Despite advancements in mapping catheters, as well as electroanatomic mapping systems, success rates of VT ablation have plateaued.
- Full diastolic pathway mapping of VT can be performed in a short amount of time and has been associated with a high rate of VT termination with RF in addition to non-inducibility at study end.

What the Study Adds?

- Mapping of the full diastolic pathway is associated with a higher freedom from VT recurrence as compared to partial mapping or substrate modification.
- Full diastolic pathway recording of VT can be achieved in a large proportion of patients.
- Patients in whom incomplete diastolic activity is tracked on the endocardial surface, epicardial mapping should be considered as a first line indication in order to identify the missing link to record the entire diastolic pathway.

American Heart Association.
Circulation: Arrhythmia and Electrophysiology

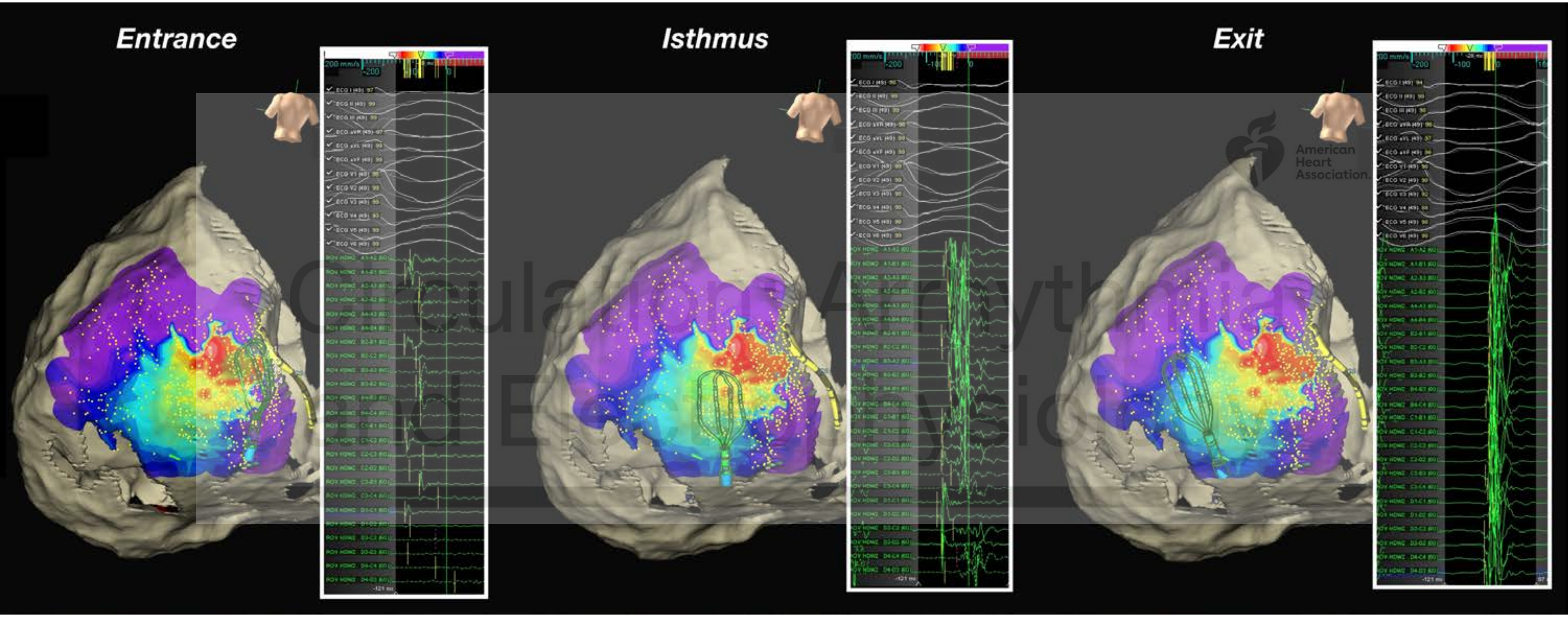

Circulation: Arrhythmia and Electrophysiology
American Heart Association.

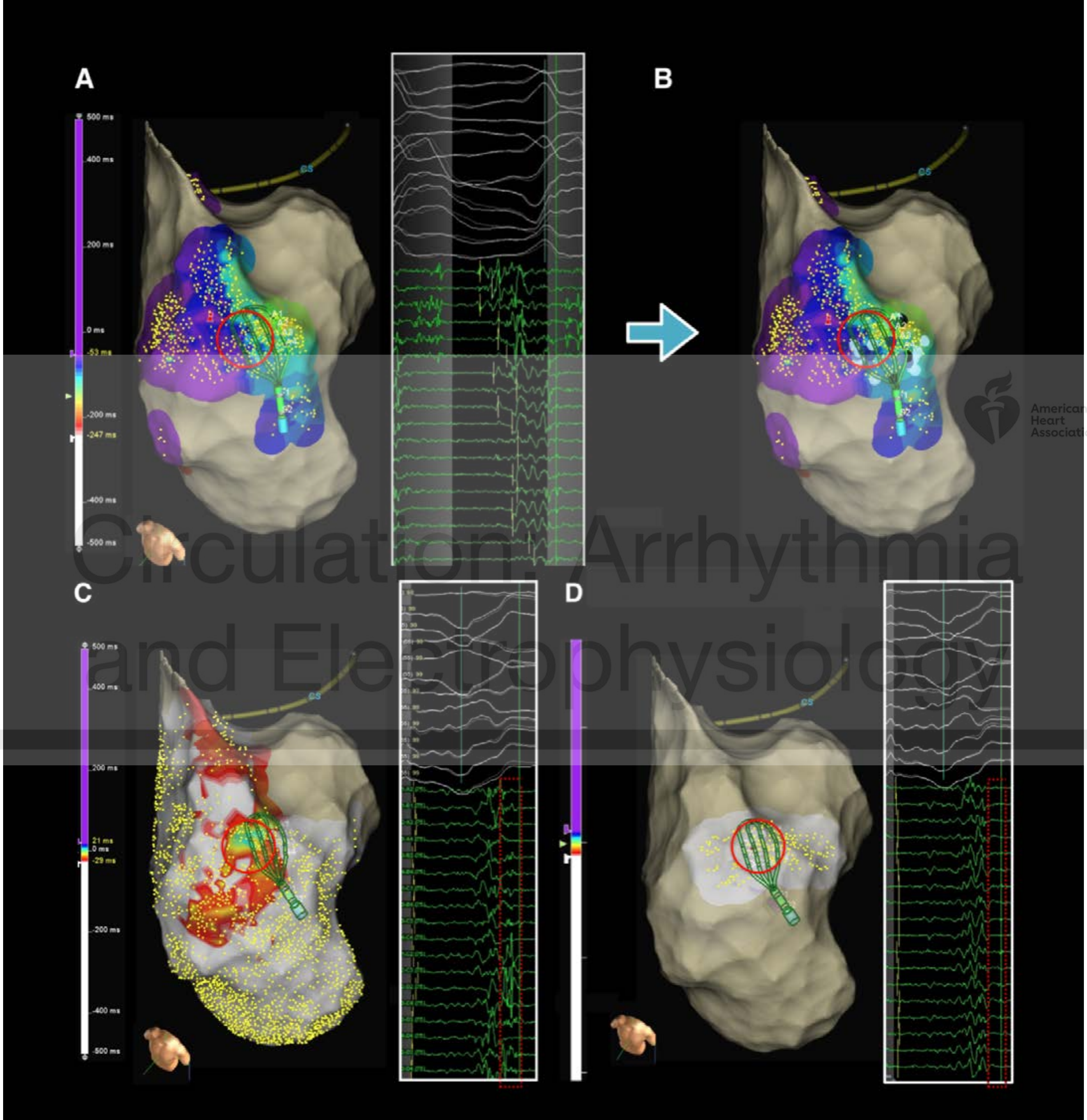

Circulation: Arrhythmia and Electrophysiology
American Heart Association.

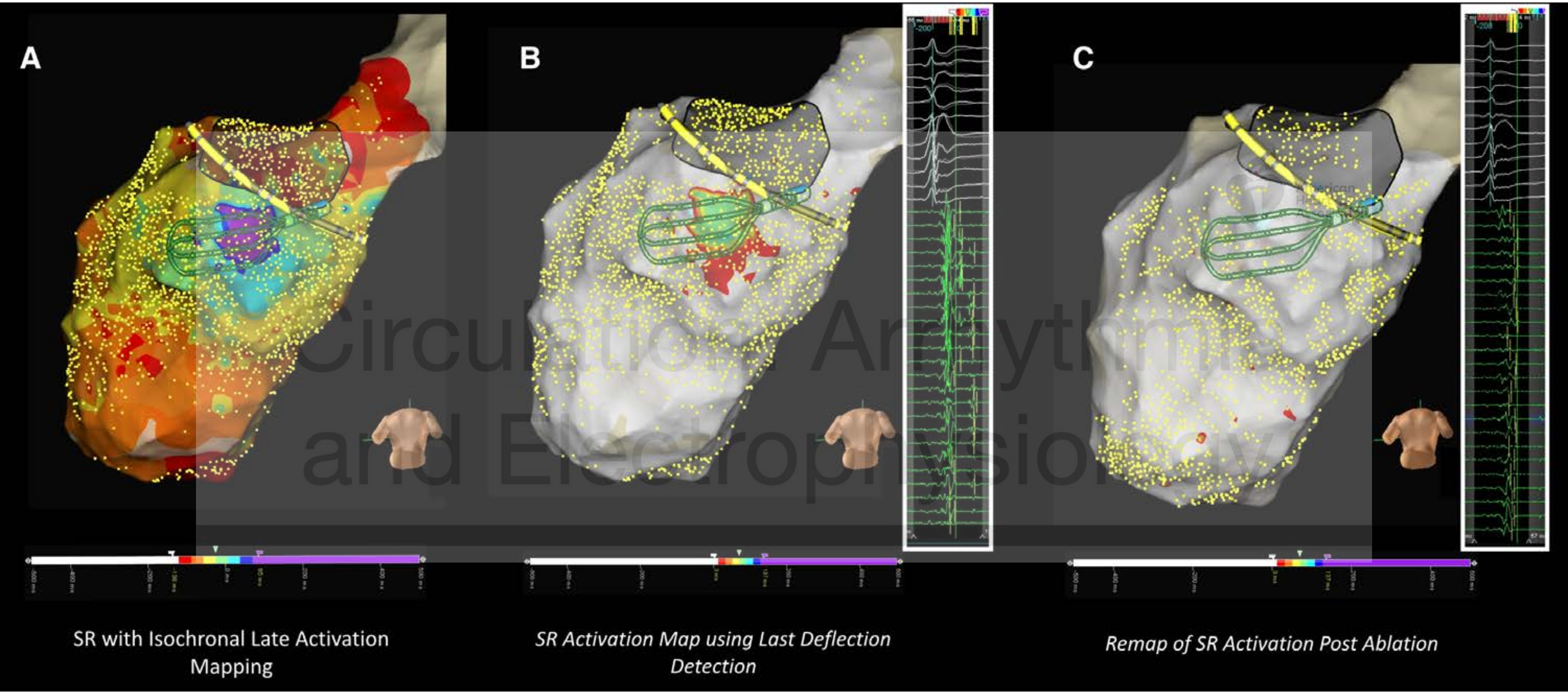

Circulation: Arrhythmia and Electrophysiology

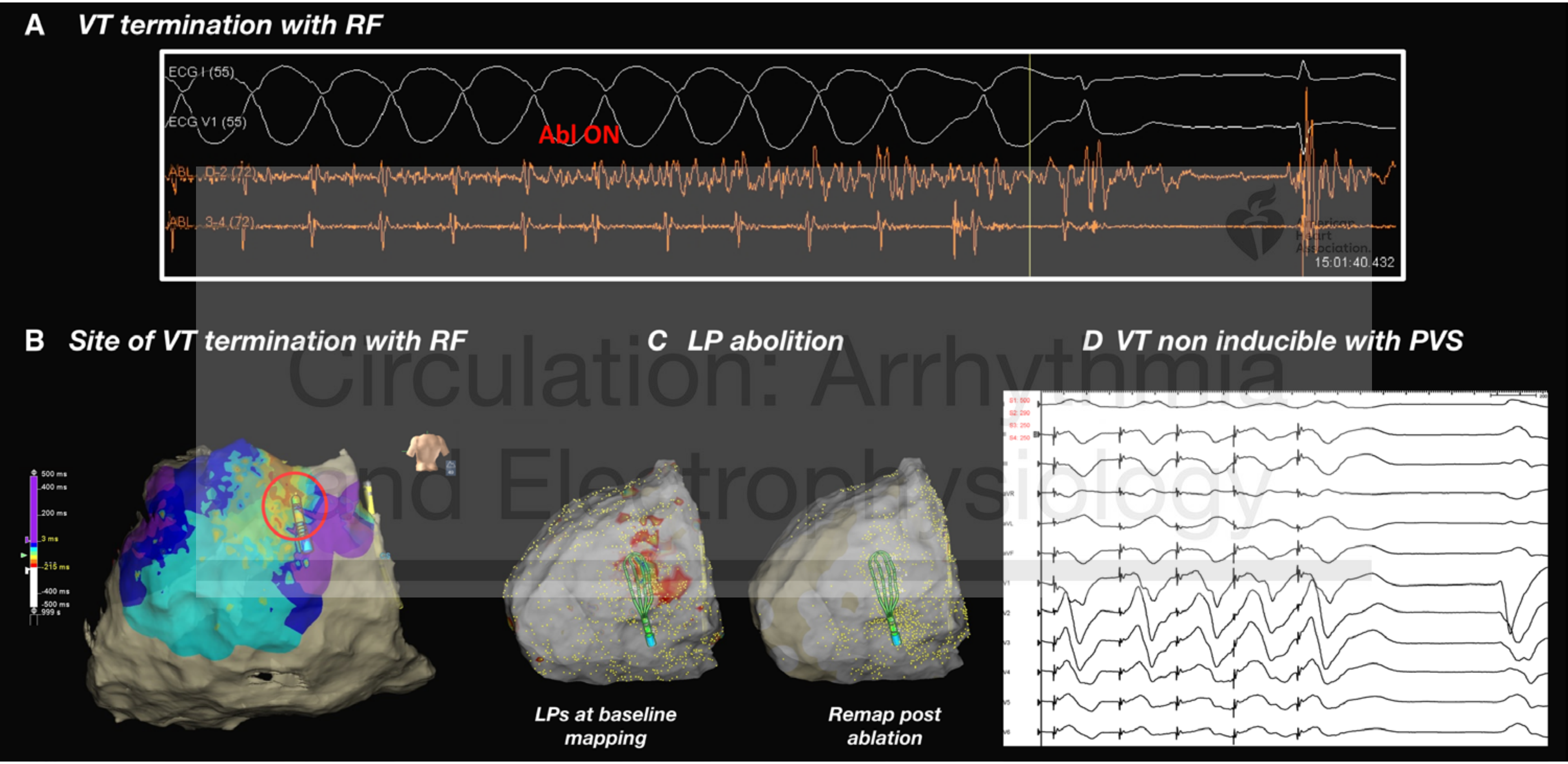

Circulation: Arrhythmia and Electrophysiology

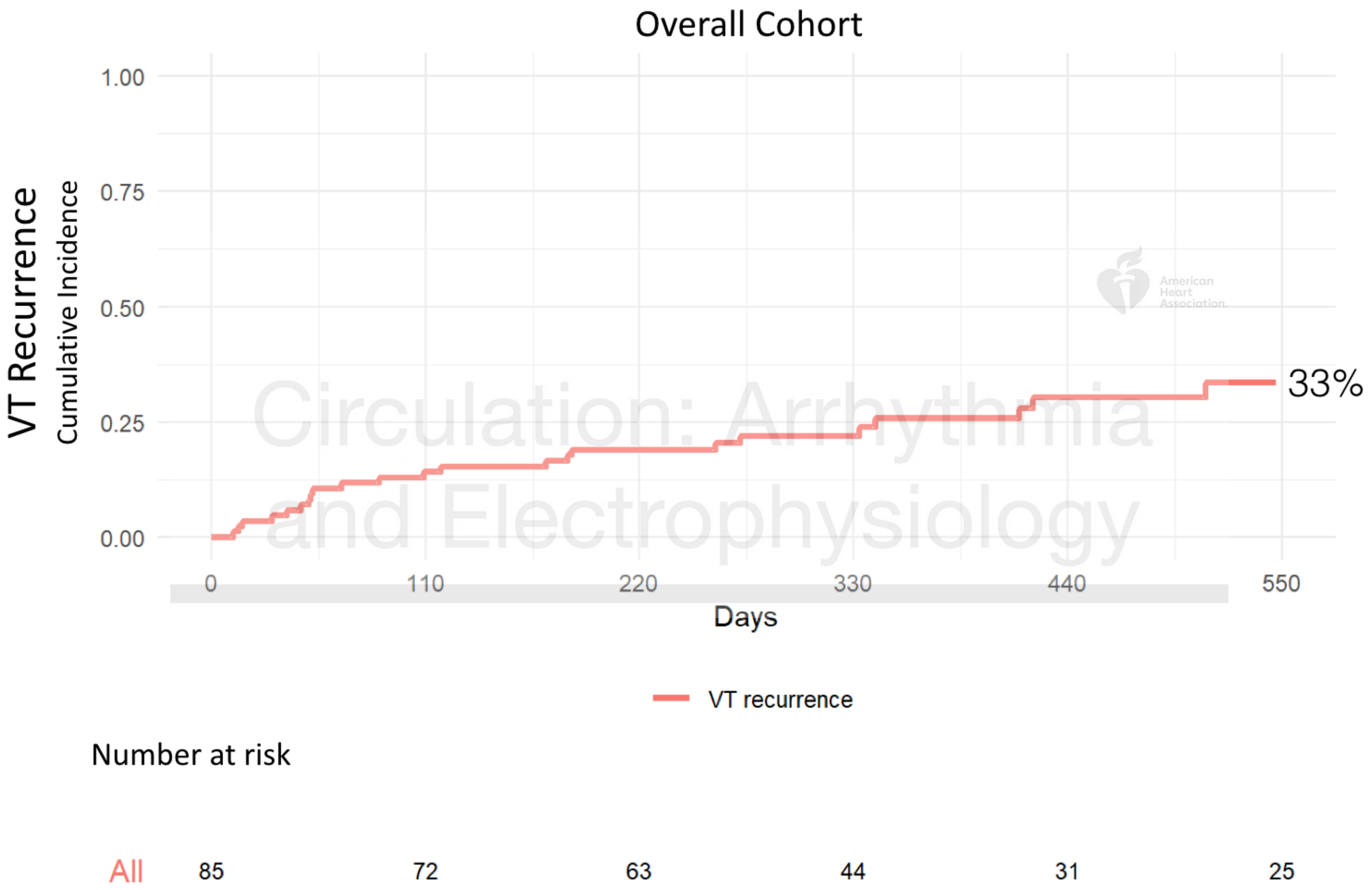

Circulation: Arrhythmia and Electrophysiology

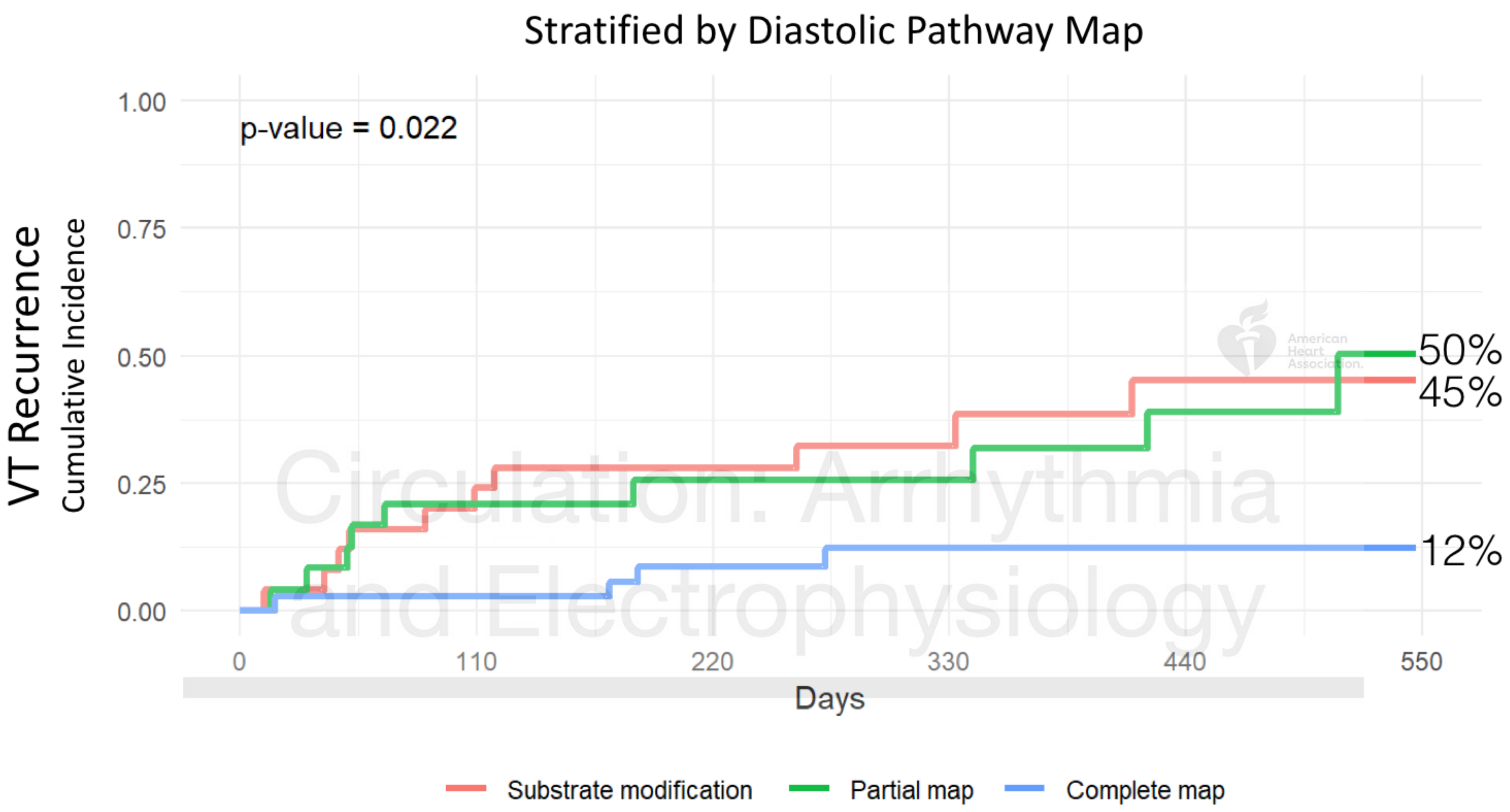

Circulation: Arrhythmia and Electrophysiology
American Heart Association.

Number at risk

	0	110	220	330	440	550
Substrate modification	25	20	18	13	9	7
Partial map	24	18	15	11	9	7
Complete map	36	34	30	20	13	11

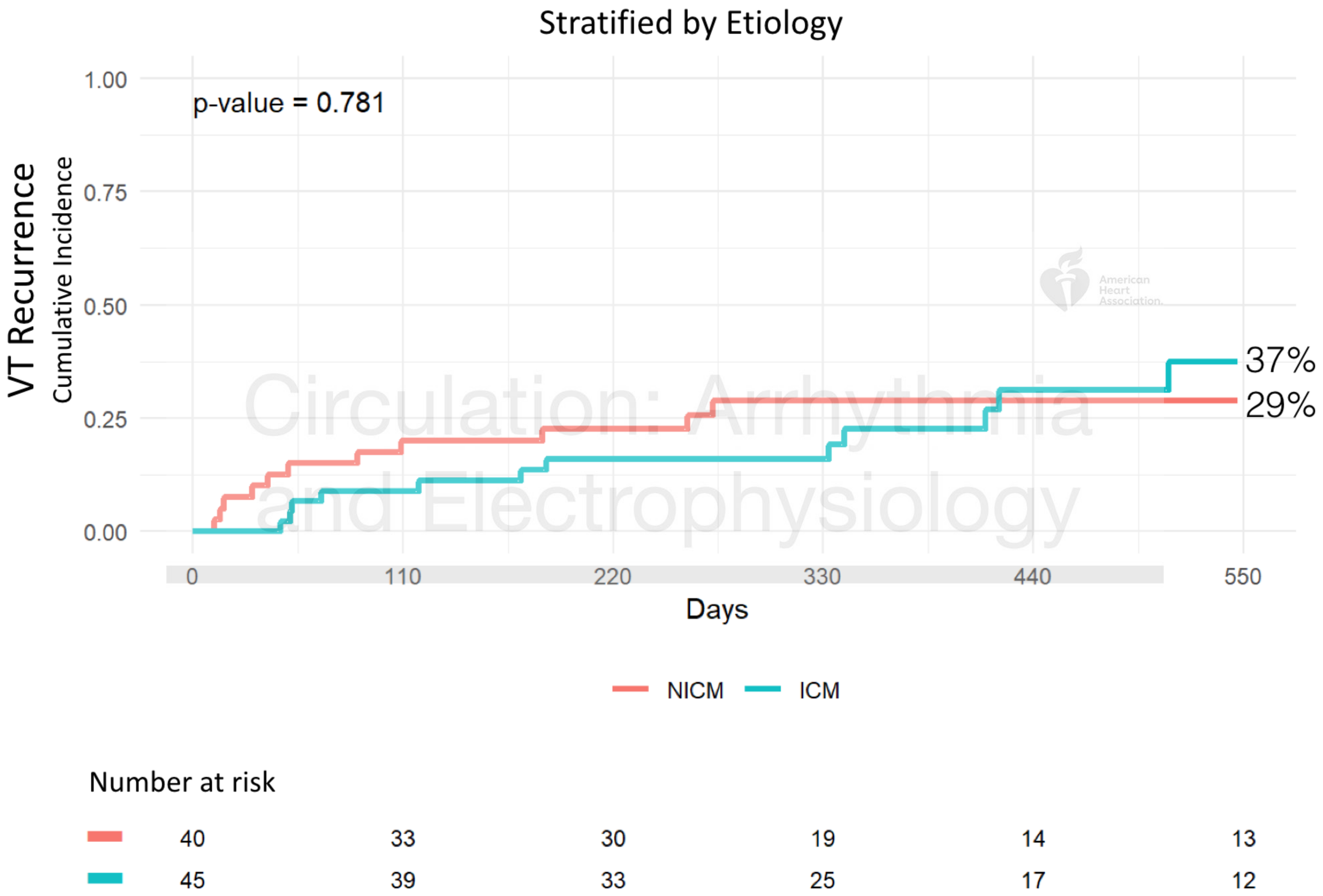

American Heart Association.
Circulation: Arrhythmia and Electrophysiology

Graphic Abstract

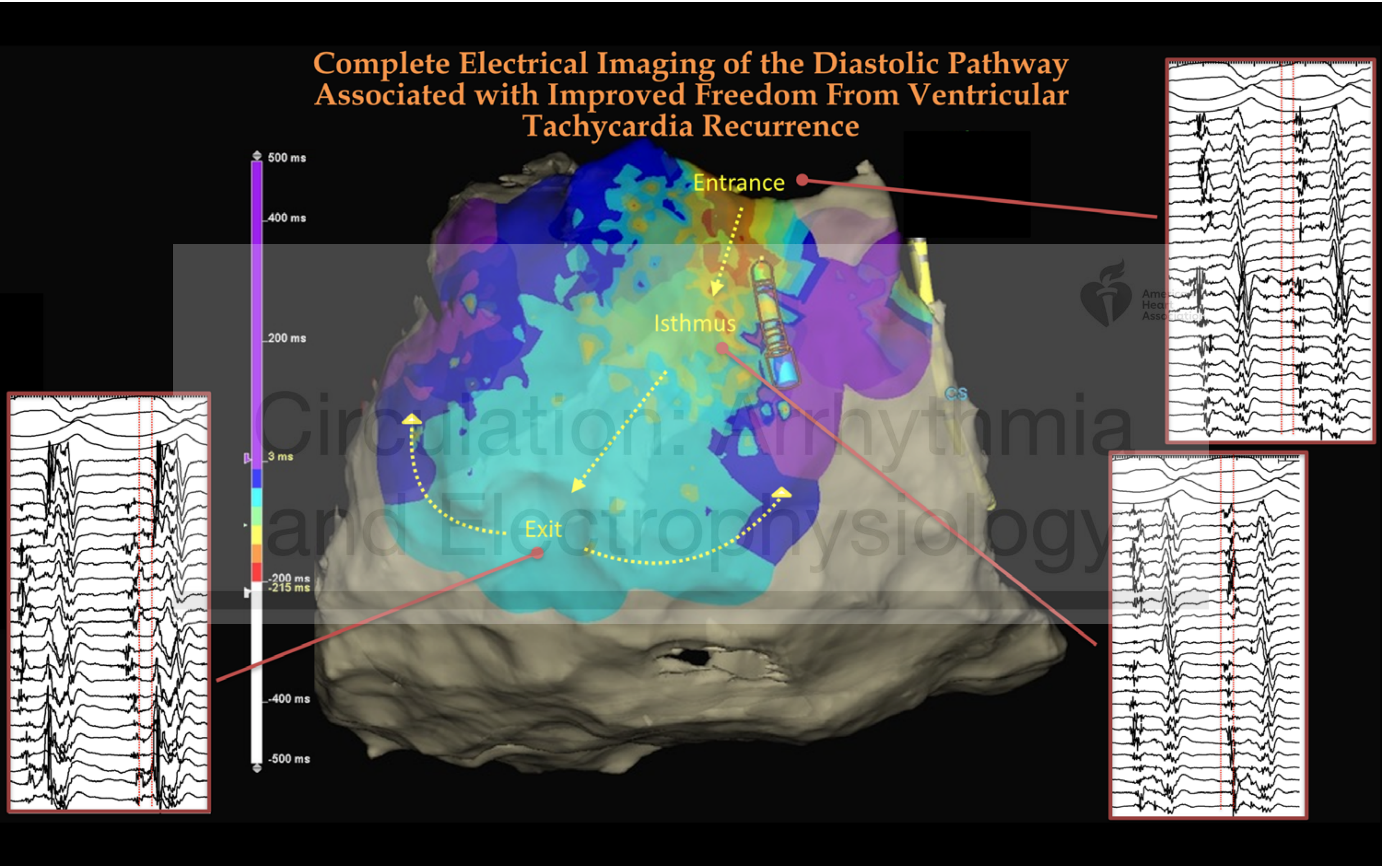

Supplement: Supplementary file 3 [file hae-13-e008651-s003.pdf]
