# Supplementary figures and images for: Complete Electroanatomic Imaging of the Diastolic Pathway Is Associated With Improved Freedom From Ventricular Tachycardia Recurrence
Source: Circ Arrhythm Electrophysiol. 2020 Jul 28;13(9):e008651. doi: 10.1161/CIRCEP.120.008651 (PMC7495983; doi:10.1161/CIRCEP.120.008651)

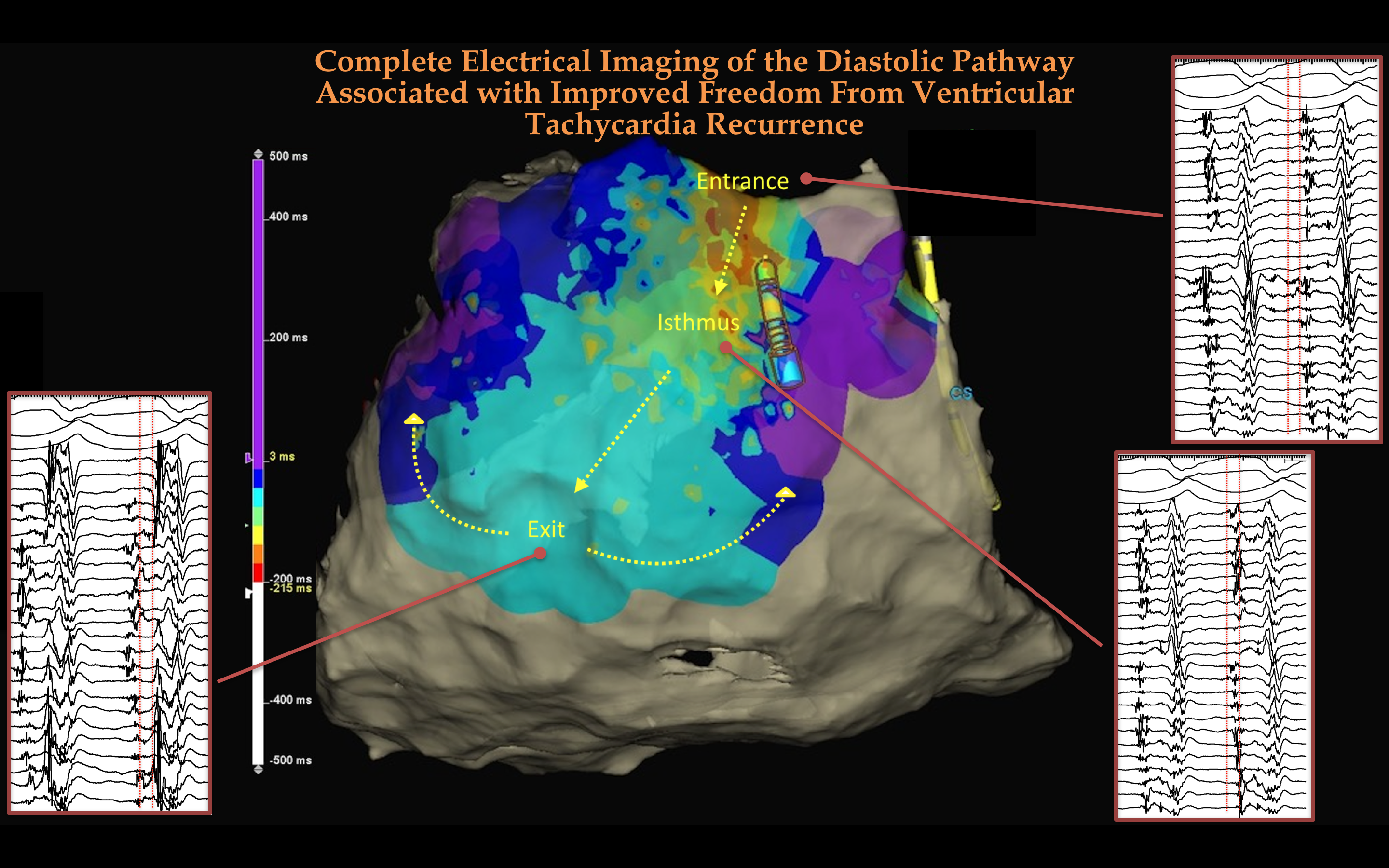

Supplement: Supplementary file 4 [file hae-13-e008651-s004.tif]
